# Cholestasis impairs gut microbiota development and bile salt hydrolase activity in preterm neonates

**DOI:** 10.1080/19490976.2023.2183690

**Published:** 2023-02-26

**Authors:** Lauren E. Lynch, Amy B. Hair, Krishnakant G. Soni, Heeju Yang, Laura A. Gollins, Monica Narvaez-Rivas, Kenneth D. R. Setchell, Geoffrey A. Preidis

**Affiliations:** a Division of Gastroenterology, Hepatology & Nutrition, Department of Pediatrics, Baylor College of Medicine and Texas Children’s Hospital, Houston, TX, USA; b Division of Neonatology, Department of Pediatrics, Baylor College of Medicine and Texas Children’s Hospital, Houston, TX, USA; c Division of Pathology and Laboratory Medicine, Cincinnati Children’s Hospital Medical Center, Cincinnati, OH, USA; d Department of Pediatrics, University of Cincinnati College of Medicine, Cincinnati, OH, USA

**Keywords:** Microbiome, cholestasis, bile acids, neonate, growth, premature infant, ursodeoxycholic acid, bile salt hydrolase

## Abstract

Cholestasis refers to impaired bile flow from the liver to the intestine. In neonates, cholestasis causes poor growth and may progress to liver failure and death. Normal bile flow requires an intact liver-gut-microbiome axis, whereby liver-derived primary bile acids are transformed into secondary bile acids. Microbial bile salt hydrolase (BSH) enzymes are responsible for the first step, deconjugating glycine- and taurine-conjugated primary bile acids. Cholestatic neonates often are treated with the potent choleretic bile acid ursodeoxycholic acid (UDCA), although interactions between UDCA, gut microbes, and other bile acids are poorly understood. To gain insight into how the liver-gut-microbiome axis develops in extreme prematurity and how cholestasis alters this maturation, we conducted a nested case-control study collecting 124 stool samples longitudinally from 24 preterm infants born at mean 27.2 ± 1.8 weeks gestation and 946 ± 249.6 g, half of whom developed physiologic cholestasis. Samples were analyzed by whole metagenomic sequencing, *in vitro* BSH enzyme activity assays optimized for low biomass fecal samples, and quantitative mass spectrometry to measure the bile acid metabolome. In extremely preterm neonates, acquisition of the secondary bile acid biosynthesis pathway and BSH genes carried by *Clostridium perfringens* are the most prominent features of early microbiome development. Cholestasis interrupts this developmental pattern. BSH gene abundance and enzyme activity are profoundly reduced in cholestatic neonates, resulting in decreased quantities of unconjugated bile acids. UDCA restores total fecal bile acid levels in cholestatic neonates, but this is due to a 522-fold increase in fecal UDCA. A majority of bile acids in early development are atypical positional and stereo-isomers of bile acids. We report novel associations linking isomeric bile acids and BSH activity to neonatal growth trajectories. These data highlight deconjugation of bile acids as a key microbial function that is acquired in early neonatal development and impaired by cholestasis.

## Introduction

Preterm birth, a live birth occurring before 37 weeks of gestation, affects 11% of global births and contributes to more deaths in children under 5 years of age than any other cause.^1^ Preterm infants are at high risk of sepsis, necrotizing enterocolitis (NEC), feeding difficulties, and liver disease^2–4^ due to structural and functional immaturity of the gastrointestinal tract. Cholestasis, impaired bile flow from the liver to the intestine, affects 1:2,500 of all live births and 10–20% of preterm births.^5^ Cholestasis can have multiple etiologies, and many cases (13–30%) are idiopathic.^6^ Risk factors include prematurity, low birth weight, and parenteral nutrition.^7,8^ There are no specific treatments for neonatal cholestasis and most interventions do not address underlying causes.^9^ Ursodeoxycholic acid (UDCA) often is administered to cholestatic infants to stimulate bile flow.^7^ However, its effects are poorly understood, especially in the preterm intestine. Cholestasis can lead to poor neonatal growth and may progress to liver failure or death.^9^ Thus, there is an urgent need to understand how cholestasis impacts the developing neonate, specifically extremely preterm infants who are at high risk of mortality.

Synthesized in the liver from cholesterol, bile acids provide the primary driving force for the production and secretion of bile, are critical for the digestion and absorption of dietary lipids,^10^ and serve as key mediators of gut-liver communication. They act as signaling molecules and tightly regulate their pool size through an efficient enterohepatic circulation.^11^ Bile acids affect the composition of the gut microbiome through bactericidal and bacteriostatic effects, and in turn, gut bacteria influence the bile acid pool.^12,13^ Microbial bile salt hydrolase (BSH) enzymes, present in many commensal microbes,^14^ perform the first step in transforming liver-derived primary bile acids into secondary bile acids, which is hydrolysis of the amino acid moiety.^13^ The gut microbiome influences the growth and development of preterm infants, and gestational age (GA) influences microbiome maturation.^15–17^ There is a knowledge gap regarding how gut bacteria and bile acids interact over time and contribute to somatic growth in extremely premature neonates. Addressing this knowledge gap could provide new therapeutic opportunities to address the comorbidities that are associated with prematurity.

We aimed to test the hypotheses that: 1) The gut microbiome and bile acid composition of extremely premature neonates develop in a predictable manner over time; 2) Cholestasis interrupts this pattern of development; and 3) UDCA treatment quantitatively modifies bile acid and microbiota profiles. In order to test these hypotheses, neonates that developed cholestasis and controls that did not develop cholestasis were matched by GA and birth weight in a single-center nested case-control study. Stool samples were collected longitudinally from birth to hospital discharge. Whole metagenomic sequencing, quantitative mass spectrometry (MS), and a unique *in vitro* BSH enzymatic activity assay optimized for low microbial biomass samples revealed that cholestasis disrupts the development of the neonatal gut microbiome and impairs bile acid deconjugation. Furthermore, we identified novel associations between bile acid biomarkers and neonatal growth. These results suggest that acquisition of microbial bile acid deconjugation activity is a critical event in early neonatal development and that the liver-gut-microbiome axis could serve diagnostic or therapeutic roles to improve neonatal outcomes.

## Results

### Patient characteristics

Twenty-four preterm neonates were included in this nested case-control study. The mean GA for all infants was 27.2 ± 1.8 weeks gestation and the mean birth weight was 946 ± 249.6 g. Baseline clinical characteristics were similar between the 12 neonates that developed cholestasis (mean peak serum conjugated bilirubin 7.0 mg/dL) during their hospitalization and the 12 matched controls that did not develop cholestasis (Table 1). Neonates in both groups had similar patterns of antibiotic use over time (Supplementary Figure S4). Additionally, parameters describing the intrauterine environment prior to birth, including the presence of maternal sepsis, preterm premature rupture of membranes, and maternal antibiotic use, were not significantly different between groups. Birth mode did not significantly affect microbiota communities (Suplementary Figure S3). Timing of serum bilirubin testing, antibiotic, and UDCA use, and fecal sample collection are depicted in Supplementary Figures S1 and S2.

**Table 1.** Baseline demographics.

	Control n = 12	Cholestatic n = 12	p-value
Gestational age (wks), *mean ± SD*	27.2 ± 1.8	27.2 ± 1.9	0.975
Birth weight (g), *mean ± SD*	968.2 ± 287.5	924.1 ± 228.4	0.682
Male, *n (%)*	6 (50.0)	6 (50.0)	1.000
Delivery by C-section, *n (%)*	11 (91.7)	9 (75.0)	0.590
SGA at birth, *n (%)*	1 (8.3)	3 (25.0)	0.590
Mother’s milk (> 90%), *n (%)*	5 (41.7)	5 (41.7)	1.000
Received prophylactic indomethacin, *n (%)*	5 (41.7)	3 (25.0)	0.667
Received surfactant, *n (%)*	11 (91.7)	9 (75.0)	0.590
Apgar Score − 5 min, *median ± IQR*	8.0 ± 1.3	8.0 ± 1.3	0.784
Received 2 doses of steroids, *n (%)*	11 (91.7)	8 (66.7)	0.317
Days of antibiotics in first 14 d of life, *median ± IQR*	3.0 ± 3.0	12.0 ± 6.5	0.148
Maternal antibiotic use, *n (%)*	12 (100)	12 (100)	1.000
Preterm premature rupture of membranes, *n (%)*	4 (33.3)	2 (16.7)	0.640
Maternal sepsis, *n* *(%)*	1 (8.3)	2 (16.7)	1.000

Values are mean ± SD (p-value from t-test), median ± IQR (p-value from Mann-Whitney test), or n (%) (p-value from Fisher’s exact test). SGA, small for gestational age.

### The extremely preterm neonatal gut microbiome develops over time

**Figure 1.**
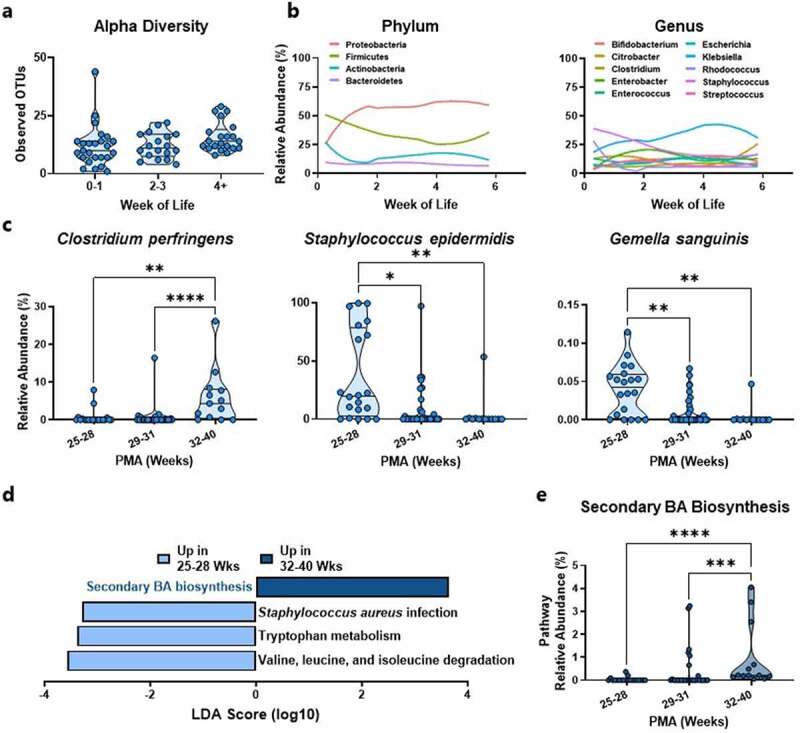
Development of the preterm gut microbiome in control neonates. (a) Observed OTUs increase with age over the first 6 weeks of life (*n* = 18–25). (b) The gut microbiome changes at both phylum and genus levels in the first 6 weeks of life (*n* = 64). (c) At the species level, *Clostridium perfringens* is most significantly increased and *Staphylococcus epidermidis* and *Gemella sanguinis* are most significantly decreased with increasing PMA (*n* = 13–31). (d) LEfSe analysis identified secondary bile acid biosynthesis as the most enriched pathway in control neonates 32–40 weeks PMA relative to control neonates 25–28 weeks PMA (*n* = 20–31). (e) The abundance of the secondary bile acid biosynthesis pathway increases with PMA (*n* = 13–31). BA, bile acid; LEfSe, linear discriminate analysis effect size; OTU, operational taxonomic unit; PMA, post-menstrual age.

To determine how gut microbial communities of extremely preterm neonates develop over time, we performed whole metagenomic sequencing on the 64 stool samples collected from the 12 neonates that did not develop cholestasis (controls) from birth to 6 weeks of age. Consistent with previous reports,^18,19^ microbial community richness increased with postnatal age (Figure 1a, *P* = 0.056). Proteobacteria and Firmicutes were the most abundant phyla throughout early development (Figure 1b). *Staphylococcus* was the most prominent genus initially, but its abundance decreased over time. In contrast, *Klebsiella* gradually increased in abundance over the first month of postnatal life (Figure 1b). Individual microbiota proportions are depicted in Supplementary Figure S3.

**** *P*<0.0001; *** *P*<0.001; ** *P*<0.01; * *P*<0.05.

In addition to these known patterns of early postnatal gut microbiota maturation, we identified new longitudinal developmental patterns among control neonates based on postmenstrual age (PMA), which is the sum of postnatal age and GA at birth (Supplementary Figure S5). Among all species, *Clostridium perfringens* increased the most in relative abundance over time (FDR adjusted global *P* = 0.01, Figure 1c). In contrast, the abundance of *Gemella sanguinis* and *Staphylococcus epidermidis* decreased with increasing PMA (FDR adjusted global *P* < 0.05, Figure 1c).

To determine how microbial functional genomic potential develops with increasing PMA, we performed linear discriminant analysis effect size (LEfSe) pathway analysis of the metagenomic signatures. The most enriched microbial pathway in stool samples from the most mature (32–40 weeks PMA) relative to the least mature (25–28 weeks PMA) neonates was secondary bile acid biosynthesis (Figure 1d). The relative abundance of this pathway increased 28-fold from early to late neonatal development (Figure 1e). In contrast, stool from the least mature neonates was enriched in microbial genes that participate in valine, leucine, and isoleucine degradation; tryptophan metabolism; and *Staphylococcus aureus* infection. Together, these data indicate that microbiome profiles from extremely preterm neonates mature with increasing PMA, and that the most prominent developmental features are the acquisition of *C. perfringens* and the secondary bile acid biosynthesis pathway.

### Cholestasis disrupts microbiome development and bile acid deconjugation

**Figure 2.**
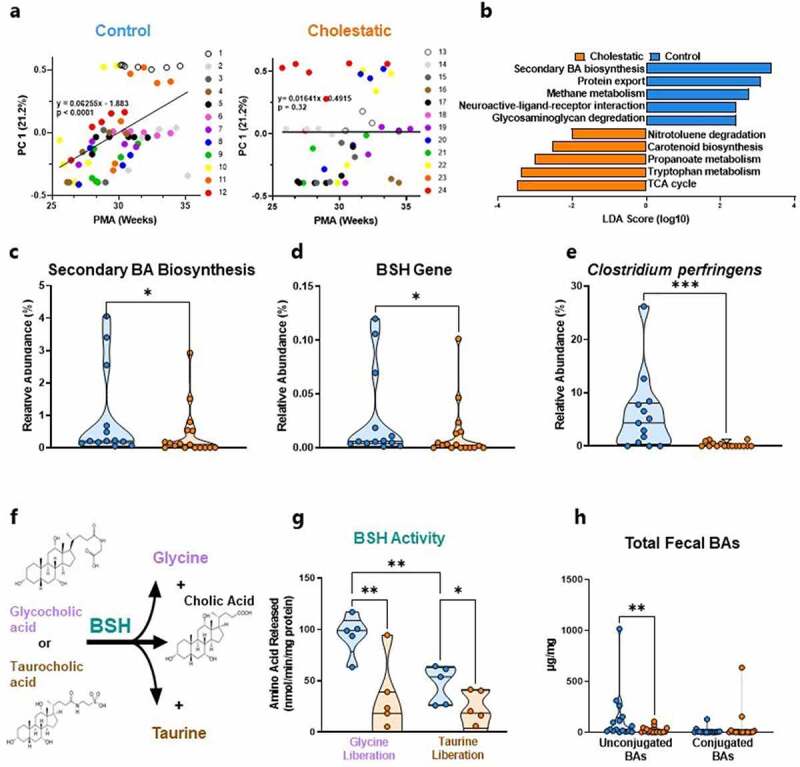
Cholestasis disrupts microbiota maturation. (a) Position on PC Axis 1 increases linearly with increasing PMA in control neonates; this pattern is absent in cholestatic neonates (*n* = 49–64). (b) LEfSe analysis identified secondary bile acid biosynthesis as the most enriched pathway in control neonates compared to cholestatic neonates 32–40 weeks PMA (*n* = 13–17). Relative abundances of (c) the secondary bile acid biosynthesis pathway, (D) the BSH gene, and (e) the BSH-carrying bacterium *Clostridium perfringens* are reduced in cholestatic neonates at 32–40 weeks PMA (*n* = 13–17). (f) Schematic of BSH activity assay. (g) Glycine deconjugation is preferred over taurine deconjugation in stool from control neonates; BSH activity is reduced in stool from cholestatic neonates (*n* = 5–6). (h) Total fecal unconjugated bile acids are reduced in cholestatic neonates, although the quantity of fecal conjugated bile acids is unchanged between groups (*n* = 16–18). BA, bile acid; BSH, bile salt hydrolase; LDA, linear discriminate analysis; LEfSe, linear discriminate analysis effect size; PC, principal coordinate; PMA, post-menstrual age.

In order to determine whether these neonatal developmental patterns are interrupted by cholestasis, we plotted developmental trajectories of gut microbiome signatures from both control and cholestatic neonates using principal coordinate (PC) analysis. PC 1, which accounted for the greatest amount of variation in the composition of the gut microbiome, was strongly associated with PMA in the control cohort (Figure 2a, *P* < 0.0001). In contrast, PC 1 was not significantly correlated with increasing PMA among cholestatic neonates (Figure 2a, *P* = 0.32), suggesting that gut microbiome development is disrupted in preterm neonates with cholestasis.

*** *P*<0.001; ** *P*<0.01; * *P*<0.05

Next, we sought to identify the specific microbial genomic pathways that cholestatic neonates failed to acquire during early development. LEfSe analysis revealed that secondary bile acid biosynthesis was the most enriched pathway in stool from the control cohort relative to the cholestatic cohort at 32–40 weeks PMA (Figures 2b and 2c, *P* = 0.04). In accord with this finding, the BSH gene, which encodes the enzyme responsible for the initial step in the microbial transformation of primary into secondary bile acids, was reduced by 55% in cholestatic neonates (Figure 2d, *P* = 0.04). We next sought to determine which species of bacteria were responsible for acquisition of the BSH gene in the control group. Within our metagenomic dataset, we identified seven BSH-carrying bacterial species, of which only *C. perfringens* was depleted in stool from cholestatic neonates relative to controls (Figure 2e, *P* = 0.0008; Supplementary Figure S6). These data indicate that cholestasis interrupts the acquisition of *C. perfringens*, the BSH gene, and the potential to synthesize secondary bile acids, all of which are prominent features of gut microbiome development in our extremely preterm control cohort.

To determine whether delayed acquisition of the BSH gene translates to impaired deconjugation of primary bile acids in cholestatic neonates, we optimized a BSH enzyme activity assay for low microbial biomass stool samples by quantifying the nanomoles of glycine or taurine liberated from glycocholic or taurocholic acid, respectively (Figure 2f). Quantifying BSH enzyme activity in stool from control neonates revealed that glycine-conjugated cholic acid was metabolized at twice the rate of taurine-conjugated cholic acid (Figure 2g, blue shapes). Given that *C. perfringens* preferentially hydrolyzes glycine over taurine conjugates,^20,21^ this result supports the notion that *C. perfringens* is the primary BSH carrier in the control cohort. As expected, BSH enzyme activity was markedly decreased in stool from cholestatic neonates (*P* < 0.05, Figure 2g). To obtain further evidence that BSH activity is impaired in cholestatic neonates, we quantified by MS all products of BSH activity, the total unconjugated bile acids, in stool from control and cholestatic neonates. Indeed, there was a median 86.4% reduction of unconjugated fecal bile acids in the cholestatic cohort (*P* = 0.0099, Figure 2h). This result is in accord with both decreased microbial BSH activity and with decreased flow of bile into the intestine in cholestasis. Taken together, these data indicate that cholestatic neonates have delayed colonization of *C. perfringens*, delayed acquisition of the BSH gene, and markedly impaired capacity to deconjugate primary bile acids.

### Fecal bile acid profiles mature over time and are altered by cholestasis

**Figure 3.**
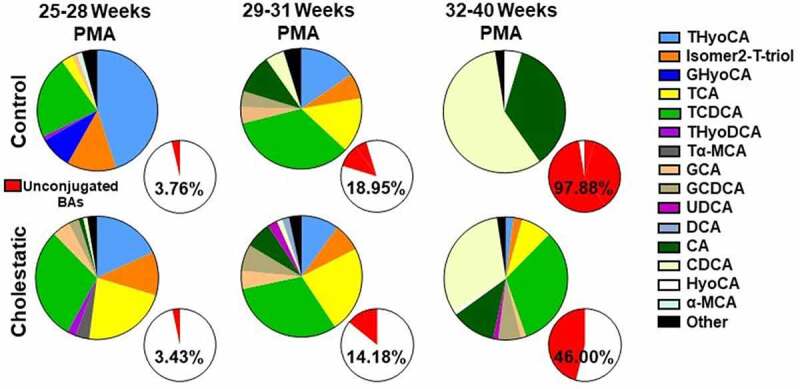
Fecal bile acid profiles are altered during cholestasis. Bile acid stereoisomers dominate in early development, then primary unconjugated bile acids increase in abundance in control neonates. Bile acid stereoisomers are less abundant and bile acid deconjugation is impaired in cholestatic neonates (excluding samples obtained during UDCA treatment) (*n* = 14–26). α-MCA, alpha-muricholic acid; CA, cholic acid; CDCA, chenodeoxycholic acid; DCA, deoxycholic acid; GCA, glycocholic acid; GCDCA, glycochenodeoxycholic acid; GHyoCA, glycohyocholic acid; HyoCA, hyocholic acid; Isomer2-T-Triol, taurine conjugate of an unidentified trihydroxy-cholanoic acid; PMA, post-menstrual age; Tα-MCA, tauro-α-muricholic acid; TCA, taurocholic acid; TCDCA, taurochenodeoxycholic acid; THyoCA, taurohyocholic acid; THyoDCA, taurohyodeoxycholic acid; UDCA, ursodeoxycholic acid.

Based on our finding that acquisition of the capacity for secondary bile acid biosynthesis was the most prominent feature of gut microbiome development in extremely preterm neonates, we sought to determine whether fecal bile acid profiles mature in parallel during the same developmental period. Thus, we quantified concentrations of individual and total fecal bile acids from 124 samples using MS based on previously described methods with modifications.^22^ At the earliest developmental stage (25–28 weeks PMA), approximately 67% of fecal bile acids identified in the extremely preterm control cohort were positional and stereoisomers of trihydroxy-cholanoic acids that were 6-hydroxylated (Figure 3). These isomers typically are present in very low levels in healthy adult feces^22^ but are a common feature of bile acid synthesis in early life.^23–25^ Consistent with previous reports^26,27^ and with our finding that BSH genes are acquired during early neonatal development, fecal unconjugated bile acids became increasingly abundant over time, accounting for only 4% of all bile acids at 25–28 weeks to 98% of the bile acids by 32–40 weeks PMA. Thus, fecal bile acid profiles develop over time and in accordance with acquisition of microbial BSH in extremely preterm neonates.

Cholestasis altered these bile salt developmental trajectories in two key respects (Figure 3). First, cholestatic neonates not given UDCA treatment had decreased proportions of 6-hydroxylated cholanoic acid isomers (29.6% vs. 66.9%) relative to control neonates at 25–28 weeks PMA. Interestingly, this difference was noted prior to the onset of cholestasis in most cases, suggesting that these isomers could potentially predict outcomes. Second, the majority of total bile acids quantified in stool from cholestatic neonates not given enteral UDCA remained conjugated (54% vs 2% in control neonates) at 32–40 weeks PMA (Figure 3), providing further evidence of impaired acquisition of bile acid deconjugation activity.

### Ursodeoxycholic acid treatment modifies the bile acid pool and microbiota composition

**Figure 4.**
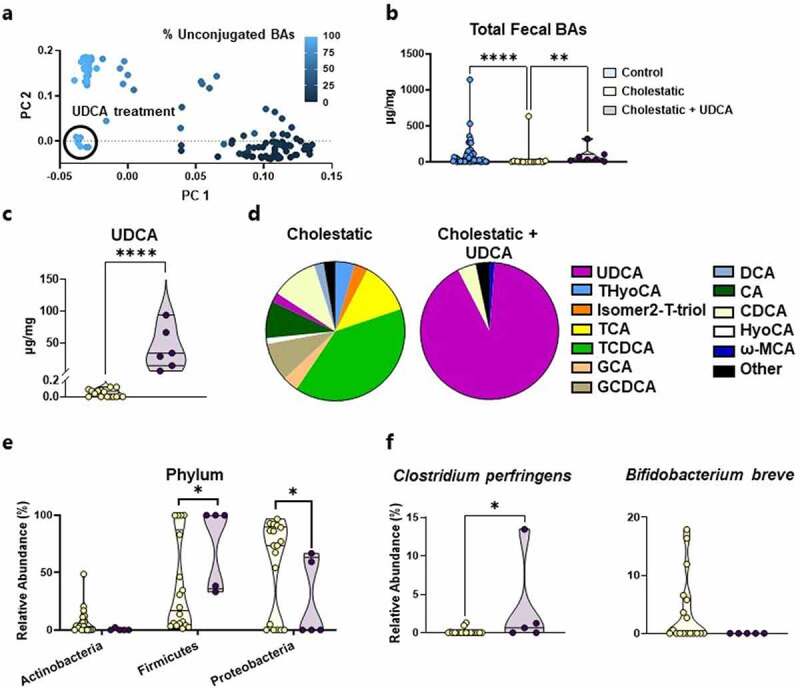
UDCA treatment alters microbiota and fecal bile acid profiles. (a) PC analysis of bile acid quantification reveals that stools obtained during UDCA treatment cluster distinctively (*n* = 124). (b) UDCA treatment restores fecal bile acid concentrations to those of control infants 29–40 weeks PMA (*n* = 7–20). (c) There are higher concentrations of UDCA in treated samples compared to untreated samples from cholestatic neonates 29–40 weeks PMA (*n* = 7–20). (d) UDCA treatment shifts the fecal bile acid pool to predominately UDCA in neonates 29–40 weeks PMA (*n* = 7–20). (e) UDCA administration increases the relative abundance of Firmicutes and decreases the relative abundance of Proteobacteria in infants 29–40 weeks PMA (*n* = 5–20). (F) BSH-carrying *Clostridium perfringens* is increased with UDCA treatment, while BSH-carrying *Bifidobacterium breve* is decreased with UDCA treatment in cholestatic infants 29–40 weeks PMA (*n* = 5–20). BA, bile acid; CA, cholic acid; CDCA, chenodeoxycholic acid; DCA, deoxycholic acid; GCA, glycocholic acid; GCDCA, glycochenodeoxycholic acid; HyoCA, hyocholic acid; Isomer2-T-Triol, taurine conjugate of an unidentified trihydroxy-cholanoic acid; ω-MCA, omega-muricholic acid; PC, principal coordinate; TCA, taurocholic acid; TCDCA, taurochenodeoxycholic acid; THyoCA, taurohyocholic acid; UDCA, ursodeoxycholic acid.

Cholestatic neonates often are treated with enteral UDCA to stimulate bile flow, although the impact of this drug on intestinal bile acid composition and gut microbial communities is unclear. In the present study, 5 of the 12 cholestatic neonates received UDCA; the remaining 7 were not treated due to enteral feeding intolerance. There were no significant differences in baseline characteristics between enrolled infants treated with UDCA, untreated cholestatic infants, and control infants (Supplementary Table S3). To understand how UDCA affects fecal bile acid profiles, we generated PC plots based on our quantitative bile acid data from all 124 fecal samples. Samples from cholestatic infants on UDCA therapy clustered separately from all other samples (Figure 4a). None of the cholestatic infants were treated with UDCA from 25 to 28 weeks PMA, thus further analyses examined samples from 29 to 40 weeks PMA. As expected, given that cholestasis represents a lack of bile flow into the intestine, total fecal bile acid concentrations were reduced in cholestasis (Figure 4b, *P* = 0.0002). Interestingly, although total fecal bile acid levels were much higher with UDCA treatment, this increase reflects unabsorbed drug. UDCA is relatively insoluble and absorbed in the proximal jejunum by nonionic passive diffusion, unlike conjugated bile acids that require the ileal bile acid transporter for reabsorption.^28,29^ Stool from cholestatic neonates on UDCA therapy had 522-fold higher concentrations of UDCA compared to untreated cholestatic neonates (Figure 4c, *P* < 0.0001). Indeed, UDCA accounted for 90% of total fecal bile acids among cholestatic neonates treated with UDCA (Figure 4d). Taken together, these data indicate that UDCA treatment dramatically alters fecal bile acid composition via the presence of unabsorbed drug.

**** *P*<0.01; ** *P*<0.01; * *P*<0.05

UDCA treatment also altered the fecal microbiota. At the phylum level, the relative abundance of Firmicutes increased, while the relative abundance of Proteobacteria decreased (Figure 4e, *P* < 0.05) among cholestatic neonates receiving UDCA compared to untreated cholestatic neonates. At the species level, the relative abundance of two BSH carriers was altered by UDCA. *C. perfringens* was enriched (Figure 4f, *P* = 0.04) and *Bifidobacterium breve* was depleted (Figure 4f, *P* = 0.13) in the stool of UDCA recipients. The loss of *B. breve* could explain why total BSH gene abundance did not differ significantly between cholestatic infants treated with UDCA and those untreated (Supplementary Figure S7). These data indicate that the dramatic fecal bile acid composition changes attributed to UDCA therapy also are associated with microbial community changes.

### Associations between microbial bile acid transformations and growth

**Figure 5.**
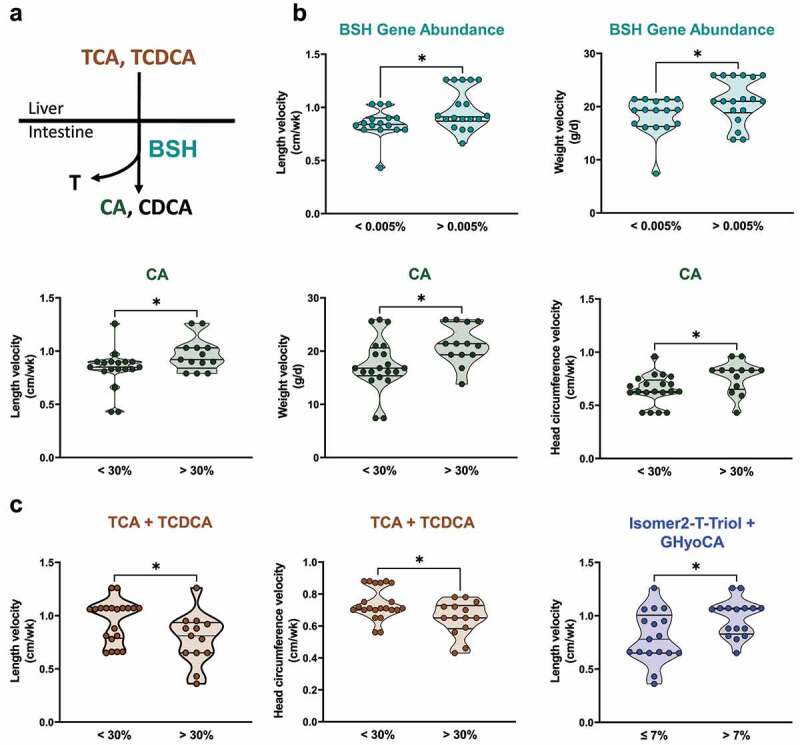
Fecal bile acids and BSH are associated with neonatal growth outcomes. (a) Schematic of BSH transformation of TCA and TCDCA into CA and CDCA, respectively. (b) In neonates 32–40 weeks PMA, the relative proportions of the BSH gene and of fecal cholic acid are associated positively with growth (*n* = 12, all groups). (c) In neonates 25–28 weeks PMA, the proportion of combined fecal TCA and TCDCA is associated negatively with growth, and the proportion of combined fecal Isomer2-T-Triol and GHyoCA is associated positively with growth (*n* = 12, all groups). BSH, bile salt hydrolase; CA, cholic acid; CDCA, chenodeoxycholic acid; GHyoCA, glycohyocholic acid; Isomer2-T-Triol, taurine conjugate of an unidentified trihydroxy-cholanoic acid; PMA, post-menstrual age; TCA, taurocholic acid; TCDCA, taurochenodeoxycholic acid.

The significance of delayed acquisition of microbial bile salt deconjugation activity is not yet known. In an exploratory analysis within our cohort, we identified novel associations between markers of microbial bile acid transformations (Figure 5a) and neonatal growth. Neonates with fecal BSH gene abundance > 0.005% at 32–40 weeks PMA exhibited 1.2-fold increased length and weight velocities compared to those with fecal BSH < 0.005% (Figure 5b, *P* < 0.05). In accord with increased BSH gene abundance, neonates with a fecal cholic acid composition of > 30% demonstrated increased length (mean increase 14%), weight (mean increase 18%), and head circumference (mean increase 15.8%) velocities (Figure 5b, *P* < 0.05) compared to those with fecal cholic acid ≤ 30%. These data suggest that fecal markers of increased BSH activity may be associated with increased neonatal growth.

* *P*<0.05

Finally, we sought to determine whether fecal bile acids present in the earliest developmental window (25–28 weeks PMA) might serve as markers of growth across the newborn hospitalization. Remarkably, we found that stools containing < 30% taurocholic acid and taurochenodeoxycholic acid (substrates for BSH enzymes) were associated with increased length (mean 22.1% increase) and increased head circumference (mean 13.6% increase) velocities (Figure 5c, *P* < 0.05) at 36 weeks PMA. Additionally, stools containing > 7% of two 6-hydroxylated bile acid isomers^28^ (taurine conjugate of an unidentified trihydroxy-cholanoic acid [Isomer 2-T-triol] and glycohyocholic acid [GHyoCA]) were associated with a mean 22.6% increased length velocity compared to stools containing ≤ 7% (Figure 5c, *P* < 0.05). These findings suggest that an impaired enterohepatic environment, including altered BSH activity (reflected by compositional changes in fecal bile acids) is associated with reduced neonatal growth velocity.

## Discussion

**Figure 6.**
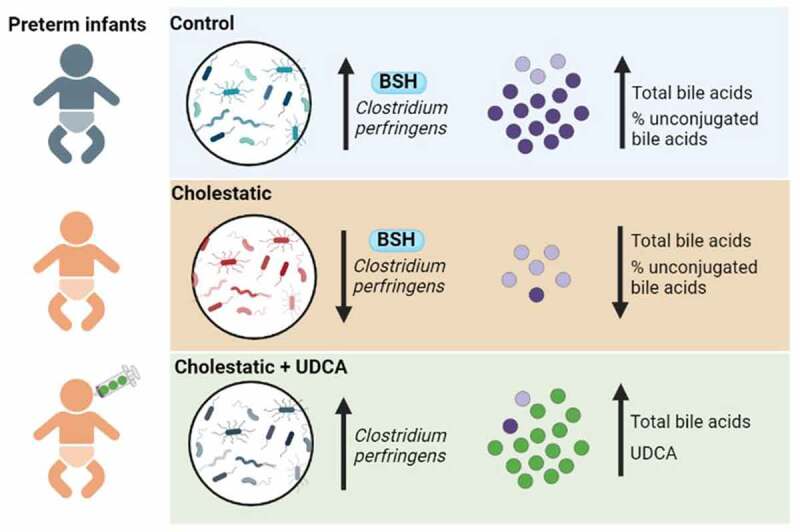
The most distinctive features of preterm gut microbiome development are increasing abundance of *Clostridium perfringens* and BSH, which increases total and relative proportions of unconjugated BAs. Cholestasis disrupts the acquisition of *C. perfringens*, BSH enzyme activity, and the capacity to form unconjugated bile acids. Enteral UDCA administration increases the abundance of *C. perfringens* and dramatically increases UDCA in the stool. BA, bile acid; BSH, bile salt hydrolase; UDCA, ursodeoxycholic acid. Created with BioRender.Com.

By characterizing fecal metagenomic profiles longitudinally among extremely preterm infants discordant for cholestasis, we have shown that gut microbiome development coincides with PMA but is compromised by cholestasis. We found that cholestasis delays the acquisition of *C. perfringens* and bile acid deconjugation activity, and that treating cholestatic neonates with UDCA increases total fecal bile acid levels by dramatically increasing the amount of fecal UDCA (Figure 6). We also identified novel associations between microbial bile acid transformation and somatic growth, with early-life bile acid metabolites potentially predicting growth trajectories throughout the neonatal intensive care unit admission.

There are relatively few published studies on the extremely preterm neonatal gut microbiome. This is in part due to the medical complexity of this fragile population, the logistical challenges related to sequencing very low microbial biomass samples that are infrequently produced (often less than one stool per week), and the challenges of profiling the bile acid metabolome in stools.^30^ Previous reports suggest that gut microbial community diversity decreases according to the degree of prematurity.^31^ During full-term development, neonates typically are colonized by potentially beneficial genera including *Bifidobacterium* and *Bacteroides*.^32^ However, premature neonates initially are colonized by members of other phyla, specifically Firmicutes followed by Proteobacteria.^33^ Several studies also have identified the genus *Clostridium* as a highly abundant member of the microbial community in preterm infants.^16,34^ We observed each of these known patterns of development in our control cohort of preterm neonates.

Our whole metagenome data extend this knowledge by identifying secondary bile acid biosynthesis and BSH genes as the most striking features of microbiome development over time. In mice, the relative abundance of the BSH gene gradually increases between birth and 28 days of life.^35^ Similarly, most healthy full-term infants are able to deconjugate bile acids by one month of age.^36^ In our cohort, we attributed the acquisition of BSH enzymes to colonization by the BSH-carrying *C. perfringens*. Often considered a pathogen due to its associations with NEC, gas gangrene, and food poisoning,^37^ others have reported that *C. perfringens* is present in the microbiota of healthy infants and children.^38,39^ A recent study of 247 *C. perfringens* isolates found increased numbers of virulence genes and higher necrotic capacity *in vitro* in isolates obtained from preterm infants with NEC compared to strains isolated from preterm infants without NEC.^40^ Therefore, the increase in *C. perfringens* over time among our control neonates, none of whom developed NEC, likely reflects colonization by less virulent or nonpathogenic strains.

There also is very limited data on how the bile acid metabolome matures over time in extremely preterm neonates.^30^ In full-term infant meconium, < 1%–16% of bile acids are unconjugated, but by 24 months 61–95% of all fecal bile acids are unconjugated.^27^ Similarly, in late preterm newborns, the majority of serum bile acids are conjugated.^41^ Precisely when unconjugated bile acids become predominant in neonates was not yet known. Our data confirm that extremely preterm newborns have mostly conjugated bile acids, and we further delineate the timeline by which the abundance of primary conjugated bile acids decreases from 96% to 2%. This timeline coincides precisely with acquisition of microbial BSH activity. Our data also highlight 6-hydroxylated bile acid isomers, which are not typically measured in most bile acid assays, as the majority of bile acids in control neonates at 25–28 weeks PMA. Gallbladder bile of fetuses between the 14^th^ and 20^th^ week of gestation contains an abundance of polyhydroxylated “atypical bile acids” with hydroxyl groups at positions C-1 and C-6.^24,42^ These bile acid metabolites result from bile acid biosynthetic pathways that involve cytochrome P450 enzymes^43^ that are active in the fetal liver^24^ and also have been observed in adults with advanced cholestatic liver disease.^44^ Not surprisingly, atypical and short-chain bile acids also are found in meconium.^25^ Therefore, it can be hypothesized that the bile acid composition in the most immature preterm neonates resembles that in fetal development, with a highly hydrophilic bile acid pool designed to protect hepatocytes from injury and to facilitate urinary excretion. Further studies are required to confirm their intriguing association with increased rate of growth in preterm infants.

We leveraged this new understanding of microbiome and bile acid maturation dynamics in extremely preterm neonates to gain new insight into how cholestasis alters this maturation process. We found that total fecal bile acid concentrations are markedly reduced in cholestasis, which is expected in the context of decreased bile flow from the liver into the intestine. However, our discovery of impaired formation of unconjugated bile acids, coinciding with delayed acquisition of *C. perfringens* and its BSH genes, may offer new insights into disease pathogenesis or therapeutic opportunities. Others have proposed BSH-carrying probiotics as a potential strategy to stimulate bile flow.^45–47^ BSH-carrying *Lactobacillus rhamnosus* GG prevents hepatic fibrosis in bile duct ligated mice by increasing fecal bile acid excretion and enhancing enterohepatic circulation via the FXR/FGF15 signaling pathways.^48^ Bile acid secretion is stimulated by similar mechanisms in a mouse model of drug-induced cholestasis by *L. rhamnosus* GG and other probiotic bacteria.^49^ Although a causal relationship between decreased BSH activity and neonatal cholestasis remains unknown, BSH-based probiotic therapy is an intriguing potential therapeutic avenue. It is notable that our cholestatic neonates were on total parenteral nutrition for a longer duration of time than the control cohort and that half of the cholestatic infants developed NEC (2/6 patients required surgery). The etiology of NEC is unknown and may be multifactorial.^50^ Future work will examine larger cohorts to study gut microbiome and bile acid interactions in relation to the development of NEC in cholestatic infants. These factors could contribute to the abnormal microbiota development in the cholestatic cohort.^51,52^

Oral UDCA therapy^28^ has been an almost universal approach to the management of neonatal cholestasis because UDCA is highly hydrophilic and generates a bicarbonate-rich hypercholeresis, but how this drug affects the liver-gut-microbiome axis is poorly understood. In our cholestatic neonates, UDCA administration altered the microbiome and increased fecal UDCA more than 500-fold to represent 90% of total fecal bile acids. In contrast, UDCA and its conjugates represent just 40% of total bile acids in duodenal contents from healthy adults given similar weight-adjusted doses,^53^ likely because it is metabolized to chenodeoxycholic and lithocholic acids.^54^ We did not detect increases in conjugated forms of UDCA in stool in part because UDCA is relatively insoluble and with a pKa of around 5 is non-ionized at intestinal pH and therefore shows limited transport to the liver by nonionic passive diffusion.^28^ In cholestasis of pregnancy, UDCA treatment increases the abundance of BSH-carrying bacteria,^55^ similar to our finding that UDCA increases BSH-carrying *C. perfringens* in cholestatic neonates. Mechanisms by which UDCA promotes the growth of BSH-carrying bacteria remain unclear.

Bile acids play a critical role in the intestine to promote the digestion and absorption of lipids.^56^ In this regard, bile acids are especially important for growth and development in the neonatal period to aid in digestion of fat, the most abundant macronutrient in their human milk diet. Preterm infants fed a human milk diet show improved gastrointestinal development, improved feeding tolerance, and decreased infection rates.^57,58^ Furthermore, preterm infants weighing ≤ 1250 grams at birth fed an exclusive human milk diet exceed targeted standards for weight gain.^59^ Optimal growth has been linked to improved neurodevelopment, thus emphasizing the importance of adequate nutrition in preterm infants.^60^ We have reported that preterm cholestatic neonates have reduced quantities of fecal bile acids. Failure to secrete bile acids into the intestine results in fat malabsorption and fat-soluble vitamin deficiency.^61^ Therefore, augmenting bile flow to improve lipid absorption in cholestatic neonates may improve growth outcomes.

Similar to neonatal cholestasis, child malnutrition decreases intestinal bile acids to further impair growth.^62^ A sub-clinical cholestasis was present in malnourished Pakistani infants aged 3–9 months; this study also identified the primary conjugated bile acid glycocholic acid, but not its unconjugated metabolite cholic acid, as a marker predicting growth faltering.^63^ Similarly, we found high proportions of primary conjugated bile acid substrates of BSH and low proportions of unconjugated products of BSH to be associated with lower head circumference, weight, and length velocities in preterm neonatal development. The potential role of these bile acids and BSH genes as predictive biomarkers must be validated in a larger independent cohort, and a potential growth-promoting causal role of these bile acids should be explored in preclinical models.

Limitations of our study include the small sample size and single-center study design. Furthermore, study participants were recruited over 4 years, which may result in environmental microbiota shifts in the neonatal intensive care unit. We performed PC analysis to confirm there was no distinct clustering of samples by year collected (Suplementary Figure S3). Also, we chose to focus on BSH as the initial and essential step in secondary bile acid biosynthesis because the bioinformatics pipeline used for this study annotates a single gene (BSH) to the Secondary Bile Acid Biosynthesis KEGG pathway. Future studies will broaden this investigation to determine whether other microbial genes (e.g., bai genes, hydroxysteroid dehydrogenases)^64,65^ and their bacterial carriers are similarly affected by cholestasis. Additionally, blood samples were not obtained from this cohort to measure serum bile acids. Future studies will analyze serum bile acids to understand how cholestasis may impact the circulating bile acid environment. Our study was not designed to determine causal links between microbial bile acid transformations and clinical outcomes; however, longitudinal sampling allowed us to describe for the first time how gut microbial communities and bile acid profiles mature in tandem in extremely preterm neonates. Additionally, by integrating metagenomic, enzyme activity, and metabolite analyses, we were able to characterize this developmental maturation in greater detail. Additional strengths include the sensitivity of our MS-based bile acid quantification, which allowed us to identify and quantify atypical bile acid metabolites in stool samples, as well as the sensitivity of our bile salt hydrolase activity assay, which facilitates the detection of quantitative changes in BSH activity from only 15 µg of fecal protein. This assay can be applied to other low microbial biomass samples including meconium.

In summary, these data lend new insights into how cholestasis affects the intestinal microbiota and bile acid composition of extremely preterm neonates. Delayed acquisition of microbial bile acid deconjugation warrants further study as a predictive marker or possibly even as a contributing factor to cholestasis and neonatal growth failure.

## Patients and methods

### Study participants and sample collection

In this nested case-control study, we selected 24 preterm infants of less than 30 weeks gestation born at Texas Children’s Hospital-Pavilion for Women (Houston, TX, United States of America). Subjects were enrolled between September 2015 and August 2019. Fecal samples were collected by the subject’s bedside nurse into sterile fecal collection containers, stored at 4°C, and transferred within 48 h into aliquots of 0.5 mL by research study personnel and stored at −80°C until analysis.

Subjects were selected from two larger observational prospective cohort studies involving preterm infants. Both studies collected serial stool samples to be used for microbiome analysis. Studies were approved by the Institutional Review Board at Baylor College of Medicine (protocols H-36828 and H-43075). All study procedures were in accordance with the ethical standards of Baylor College of Medicine. Written parental informed consent was provided for each infant enrolled in each study. Eligible study participants from Study 1^66^ included premature infants born at < 1500 g with no barriers to enteral milk feedings enrolled within the first 72 h of life. Exclusion criteria included anomalies or birth defects that precluded enteral feeding, severe perinatal hypoxia, or < 50% projected survival based on the National Institute of Child Health and Human Development Neonatal Research Network Extremely Preterm Birth Outcome Data calculator. For Study 2, eligible study participants included premature infants born between 24 and 34 weeks GA, birth weight ≤ 1250 g, and enrolled within the first 72 h of life. Infants were excluded for > 1250 g birth weight, low likelihood of survival, major congenital anomalies or clinically significant congenital heart disease, intestinal perforation, Bell’s Stage 2 or greater NEC, early transfer to another institution, or failure to achieve full enteral feedings by 28 days of life.

Cholestasis was defined by a serum conjugated bilirubin level ≥ 1 mg/dL and > 20% of the total bilirubin at any point during hospital admission. Elevated conjugated bilirubin represents impaired bile formation or flow.^9^ All neonates were screened within the first 48 h of life and again as clinically indicated (e.g., concerns for sepsis or new-onset jaundice). Each cholestatic infant was matched to a control (non-cholestatic) infant with emphasis on matching by birthweight, sex, delivery by Cesarean section, antibiotic exposure, % of feeds mother’s milk, and multiple fecal samples obtained. Anthropometrics were obtained at birth and approximately 36 weeks PMA by a trained research nurse. Growth velocities (weight, length, and head circumference) were calculated from birth to approximately 36 weeks PMA. The timing of patient fecal sampling, serum conjugated bilirubin measurements, antibiotic use, and UDCA treatment (if applicable) are shown in Supplementary Figures S1 and S2.

### Fecal microbiome analysis

Complete methodological details are presented in the STORMS^67^ (Strengthening the Organization and Reporting of Microbiome Studies) checklist (Table S1, Tables S4-S12). Briefly, whole metagenome shotgun sequencing was performed at the Alkek Center for Metagenomics and Microbiome Research at Baylor College of Medicine. Fecal samples were collected and stored at −80°C until DNA was extracted using the DNeasy 96 PowerSoil Pro QIAcube HT Kit (Qiagen) according to the manufacturer’s protocol. DNA was quantified using Qubit fluorometric quantitation prior to library preparation. Sequencing was performed on the Illumina NovaSeq S4 platform using the 150 bp paired-end read protocol (average sequencing depth = 41518559; minimum sequencing depth = 33936). Negative controls were tubes with reagents from DNA extraction and water only, and positive controls were from the MSA-2002 and MSA-1003 mock communities (ATCC). Quality filtering and trimming were performed using BBTools (sourceforge.net/projects/bbmap/; BBMap version 38.82). Trimming parameters were set to a k-mer length of 19 and a minimum Phred quality score of 25. Reads with a minimum average Phred quality score below 23 and length shorter than 50 bp after trimming were discarded. Final sample size after quality control was 113 (original total = 124). The taxonomic composition of the filtered reads was determined using the MetaPhlAn3 software package.^68^

Functional metagenomic analysis was performed with the HUMAnN3 package.^68^ Microbial genes and metabolic pathways were annotated to the Kyoto Encyclopedia of Genes and Genomes (KEGG) database.^69^ Raw data files have been submitted to the NCBI SRA database under the accession number PRJNA870588.

### Bile acid profiling by ultra-performance liquid chromatography coupled with tandem mass spectrometry (UPLC-MS/MS)

The fecal bile acid metabolome was determined by ultra-performance liquid chromatography coupled with tandem mass spectrometry (UPLC-MS/MS). Bile acid extraction was carried out on wet feces. Samples were weighed, homogenized, and sonicated and bile acids extracted based on previous methods^22,70^ using 80% methanol/water followed by chloroform/methanol (2:1, v/v). The supernatants were combined and dried under nitrogen. The dried extract was dissolved in 1 mL of methanol and a 1/20th aliquot was used for sample clean-up and bile acid extraction using a C18 solid phase cartridge. Internal standards comprising 15 stable-isotopically labeled bile acids were added to the fecal extract prior to the extraction. The bile acid final extract was reconstituted in 200 µL of methanol/water (50/50 v/v) and analyzed by UPLC-MS/MS.

Quantitative analysis of the individual major bile acids and their isomers in the fecal extracts was conducted by UPLC-MS/MS with electrospray ionization (ESI) using a Waters Xevo TQ-S triple quadruple mass spectrometer interfaced with an Aquity UPLC system (Waters Corporation, Milford, MA). Individual bile acid species were separated on a Kinetex C18 (2.6 µm, 100 × 3.0 mm) column (Phenomenex, Torrance, CA) with gradient elution consisting of mobile phase A (20% acetonitrile/water with 10 mM ammonium acetate) and mobile phase B (80% acetonitrile/water with 10 mM ammonium acetate) programmed from 5% mobile phase B to 100% mobile phase B, with a total run time of 20 min. Calibration curves used for quantification were built with 23 of the bile acid reference compounds and detected by multiple reaction monitoring (MRM) function operated with negative ion detection.

### Bile salt hydrolase activity assay

A colorimetric ninhydrin BSH activity assay optimized for stool samples was adapted from Tanaka et al. and Gopal-Srivastava et al.^20,71^ The experimental samples varied in fluidity, had low microbial and protein content, and contained insoluble particles that interfered with colorimetric estimation of glycine or taurine. Given these limitations, we optimized the following protocol for precise measurements of BSH activity. First, stool samples were dried at 37°C and dry weight was used for optimal microbial protein recovery. After suspending 300 mg dried stool samples in 3 mL suspension buffer (PBS with 10% glycerol, 5 mM DTT, and protease inhibitor cocktail), the solution was sonicated on ice for 6 cycles of 30 s on/off cycles. Sonicated stool suspensions were centrifuged at 4000 g for 10 min at 4°C. Supernatants were concentrated using Amicon Ultra-5 10K centrifugal filters. Approximately 300 µL of concentrated sample was further centrifuged at 16,000 g for 10 min at 4°C. The protein extract was stored at −80°C until use. Assay buffer (10 mM citrate buffer, 10 mM DDT; pH 5.8) was added to 15 µg of fecal protein, then 10 mM of GCA or TCA was added for a final volume of 200 µL. Negative control samples contained 200 µL of the reaction mixture with 200 µL of 15% TCA. Reactions were carried out at 37°C, and 100 µL was collected after 30 and 60 min of incubation. Immediately after sample collection, samples were mixed with an equal volume of 15% TCA and placed on ice. Samples were centrifuged (16,000 g, 4°C, 15 min) and the supernatant was collected. Ninhydrin reagent (263 µL ninhydrin, 632 µL glycerol, 105 µL 0.5 M citrate buffer; pH 5.5) was added to the samples, which were boiled for 15 min. Tubes were cooled under running water; from each tube 200 µL of volume was further diluted to 1.2 mL with water and the absorbance at 570 nm was determined. Nanomoles of glycine or taurine were calculated against a standard curve and background absorbance for each sample was subtracted using the negative control values.

### Statistical analysis

Statistical analyses were performed in GraphPad Prism (v. 9.3.0) and RStudio (v. 1.4.1106). Codes for data analysis and complete metadata can be accessed at https://github.com/laurenevelynch/Preterm_Cholestasis.git.

Community abundance and alpha diversity metrics were obtained from count data using the *phyloseq* package.^72^ Relative abundances were used for further microbiota analyses and calculated using the total-sum scaling (TSS) technique. Kruskal-Wallis (KW) tests with FDR adjustment were applied to the dataset of control infants to determine differences in species relative abundance. LEfSe was performed to detect differentially abundant pathways with an effect size cut-off of 2.0 and KW *P* < 0.05.^73^ Differences in pathway and species abundances by PMA in control infants were analyzed with KW tests followed by Dunn’s multiple comparisons test. PC analysis with Bray-Curtis dissimilarity was performed in the following manner using the *vegan* package.^74^ First, the PC 1 axis that accounted for the greatest variation among microbiota communities from control neonates was determined. Next, this PC 1 axis was used to generate a second PC plot for cholestatic neonates based on weighted PC analysis with the wcmdscale function in *vegan*. For this second plot, samples from cholestatic neonates were assigned a weight of 0 and their position on PC 1 relative to control samples (weight = 1) was computed. Finally, the relationship between PC 1 and PMA for both groups was determined via linear regression.

All data were tested for normality, and non-parametric Mann-Whitney tests were applied for non-normally distributed data to test significance between the control and cholestatic groups at 32–40 weeks PMA. BSH activity assay data were normally distributed and assessed via ANOVA with correction for multiple comparisons by the Benjamini and Hochberg method. Bile acid relative abundance is depicted as median values in pie charts. PC coordinates for bile acid data were generated via the prcomp package in R. Total fecal bile acids between control and treatment groups were analyzed via KW tests with correction by Dunn’s. Phyla and species differences between treatment groups were assessed by Mann-Whitney tests.

Clinical characteristics were tested for normality by the Shapiro-Wilk test. Comparisons were analyzed with t-tests for normally distributed continuous variables, Mann-Whitney tests for non-normally distributed data, and Fisher’s exact test for categorical variables. For comparisons with more than two groups, ANOVA or KW tests were used. To analyze associations between microbial bile acid transformations and growth, data were visualized with histograms and inflection points within the data set were identified. Significance was assessed at the α < 0.05 level.

## Supplementary Material

Supplemental Material

## Data Availability

The data are openly available in the National Center for Biotechnology Information Sequence Read Archive under the accession number PRJNA870588.

## Abbreviations

α-MCA, alpha-muricholic acid, 3α,6β,7α-trihydroxy-5β-cholanoic acid; BSH, bile salt hydrolase; CA, cholic acid, 3α,7α,12α-trihydroxy-5β-cholanoic acid; CDCA, chenodeoxycholic acid, 3α,7α-dihydroxy-5β-cholanoic acid; DCA, deoxycholic acid, 3α,12α-dihydroxy-5β-cholanoic acid; FDR, false discovery rate; GA, gestational age; GCA, glycocholic acid, glyco-3α,7α-dihydroxy-5β-cholanoic acid; GCDCA, glycochenodeoxycholic acid, glyco-3α,7α-dihydroxy-5β-cholanoic acid; GHyoCA, glycohyocholic acid, glyco-3α,6α,7α-trihydroxy-5β-cholanoic acid; HyoCA, hyocholic acid, 3α,6α,7α-trihydroxy-5β-cholanoic acid; isomer 2-T-triol, taurine conjugate of an unidentified trihydroxy-cholanoic acid; KW, Kruskal-Wallis; LDA, linear discriminant analysis; LEfSe, linear discriminant analysis effect size; MS, mass spectrometry; NEC, necrotizing enterocolitis; ω-MCA, omega-muricholic acid, 3α,6α,7α-trihydroxy-5β-cholanoic acid; PC, principal coordinate; PMA, post menstrual age; Tα-MCA, tauro- alpha-muricholic acid, tauro-3α,6,7α-trihydroxy-5β-cholanoic acid; TCA, taurocholic acid, tauro-3α,7α,12α-trihydroxy-5β-cholanoic acid; TCDCA, taurochenodeoxycholic acid, tauro-3α,7α-dihydroxy-5β-cholanoic acid; THyoCA, taurohyocholic acid, tauro-3α,6α,7α-trihydroxy-5β-cholanoic acid; THyoDCA, taurohyodeoxychloic acid, tauro-3α,6α-dihydroxy-5β-cholanoic acid; UDCA, ursodeoxycholic acid, 3α,7α-dihydroxy-5β-cholanoic acid; UPLC-MS/MS, ultra-performance liquid chromatography coupled with tandem mass spectrometry.
