# Supplementary material for: Cholestasis impairs gut microbiota development and bile salt hydrolase activity in preterm neonates
Source: Gut Microbes. 2023 Feb 26;15(1):2183690. doi: 10.1080/19490976.2023.2183690 (PMC9980517; doi:10.1080/19490976.2023.2183690)
Supplement: Supplemental Material [file KGMI_A_2183690_SM7175.docx]

**Supplementary Data**

**
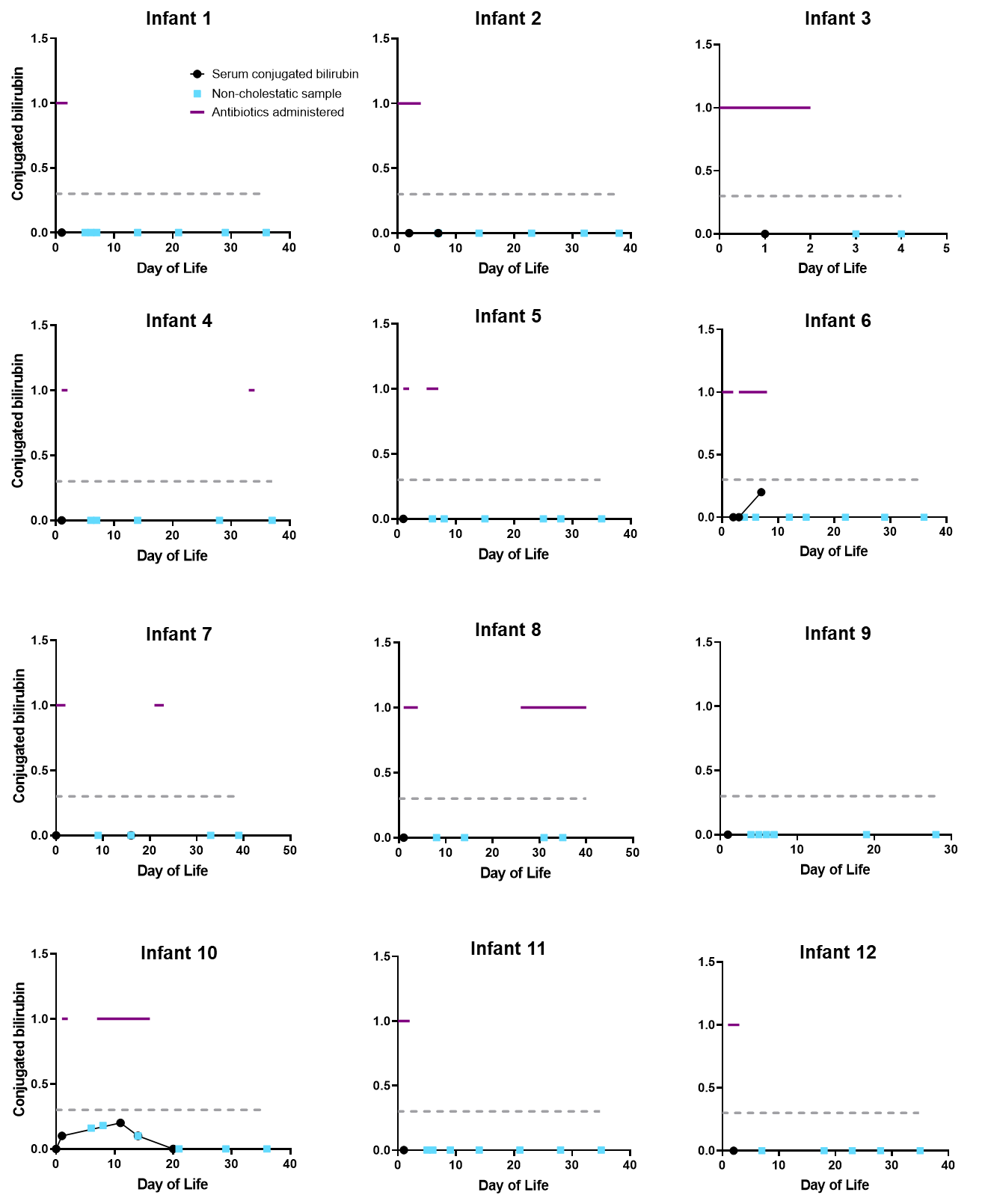
**Figure S1: Sampling schedule, antibiotic use, and serum conjugated bilirubin over time for each infant in the control cohort.


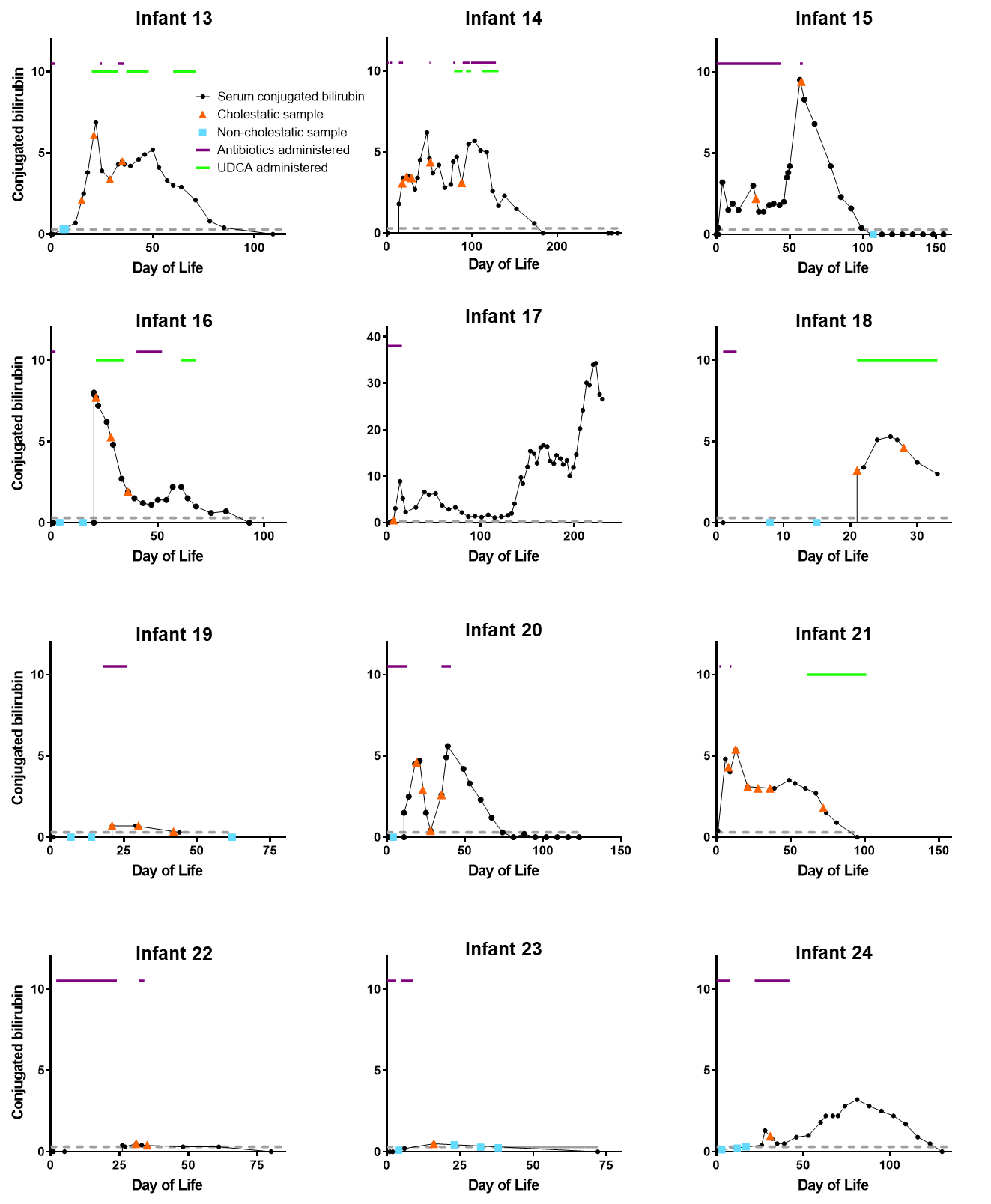


Figure S2: Sampling schedule, antibiotic use, UDCA use, and serum conjugated bilirubin over time for each infant in the cholestatic cohort. Non-cholestatic fecal samples are depicted by a turquoise square and cholestatic fecal samples are depicted by an orange triangle. UDCA, ursodeoxycholic acid.


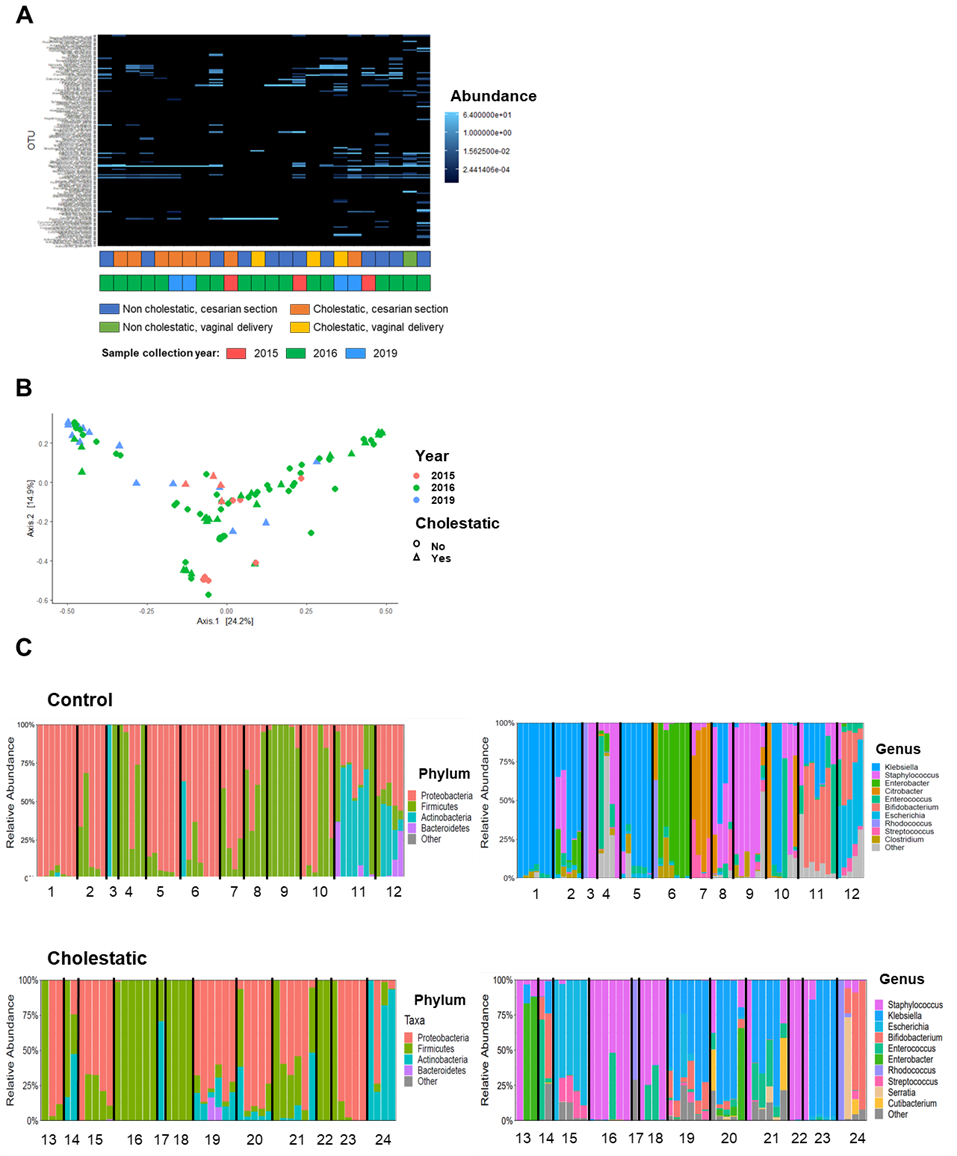


Figure S3. Individual microbiome communities do not cluster by birth mode or date of sample collection. (A) Bray-Curtis clustering and heat map visualization of species data for the first sample collected for each patient demonstrates that neither mode of birth nor collection year impact clustering (n = 24). (B) Principal coordinate analysis reveals there is no longitudinal shift in microbiota communities based on sample collection year (n = 113). (C) Individual microbiome communities at the phylum and genus levels over time (n = 113).


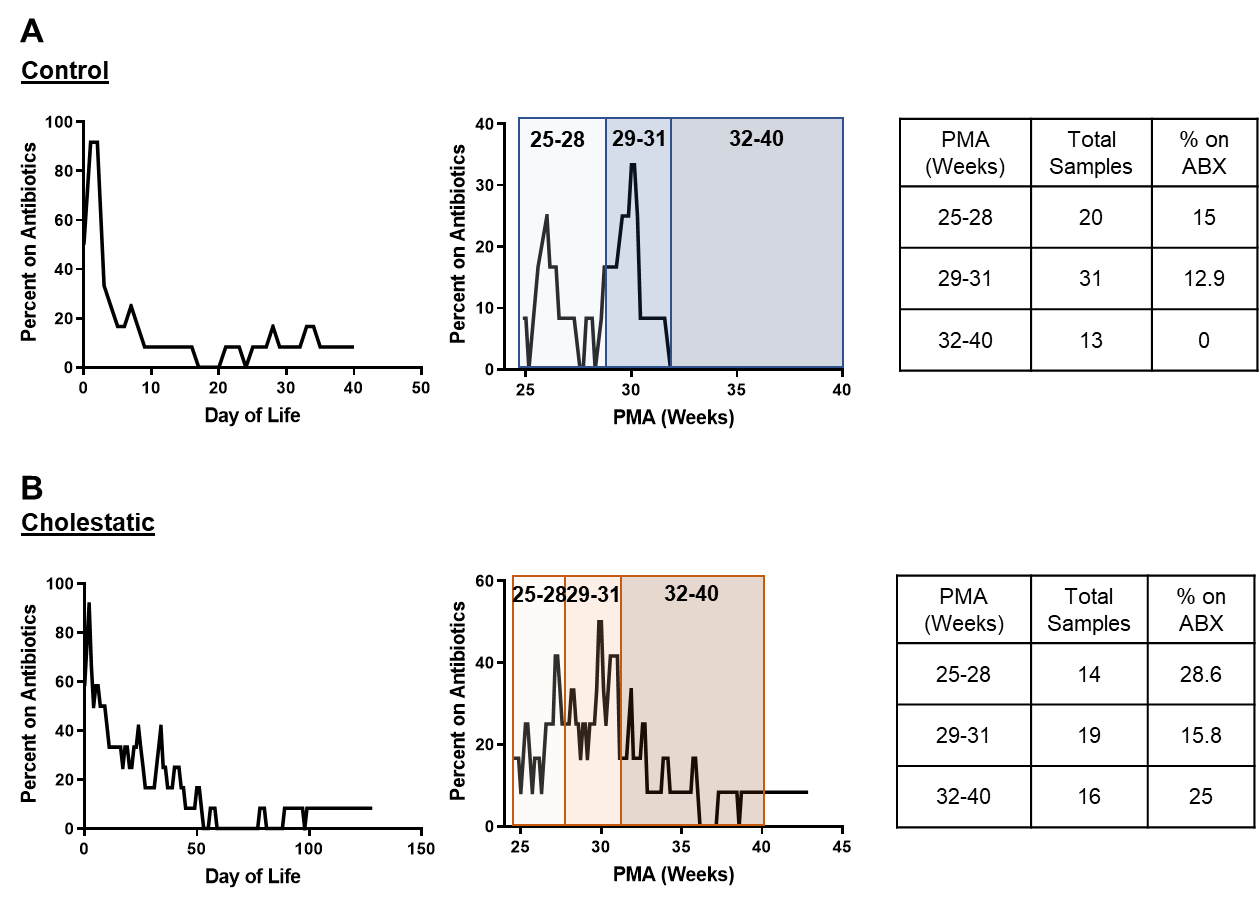


Figure S4. Antibiotic use over time in preterm control (A) and cholestatic (B) cohorts.

**
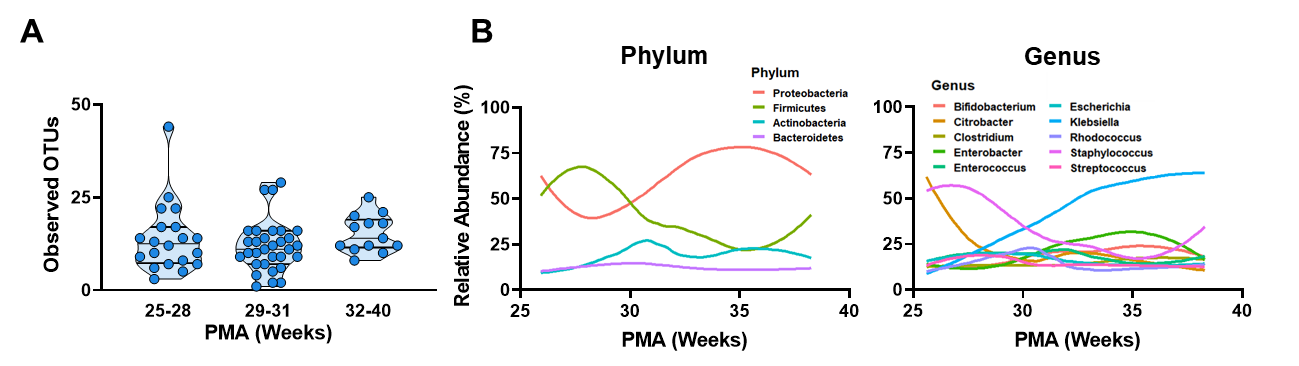
**

Figure S5. Development of the preterm gut microbiome in control neonates based on PMA. (A) Observed OTUs increase with increasing PMA (n = 13-31). (B) The gut microbiome changes at both phylum and genus levels with increasing PMA (n = 64). PMA, post-menstrual age.


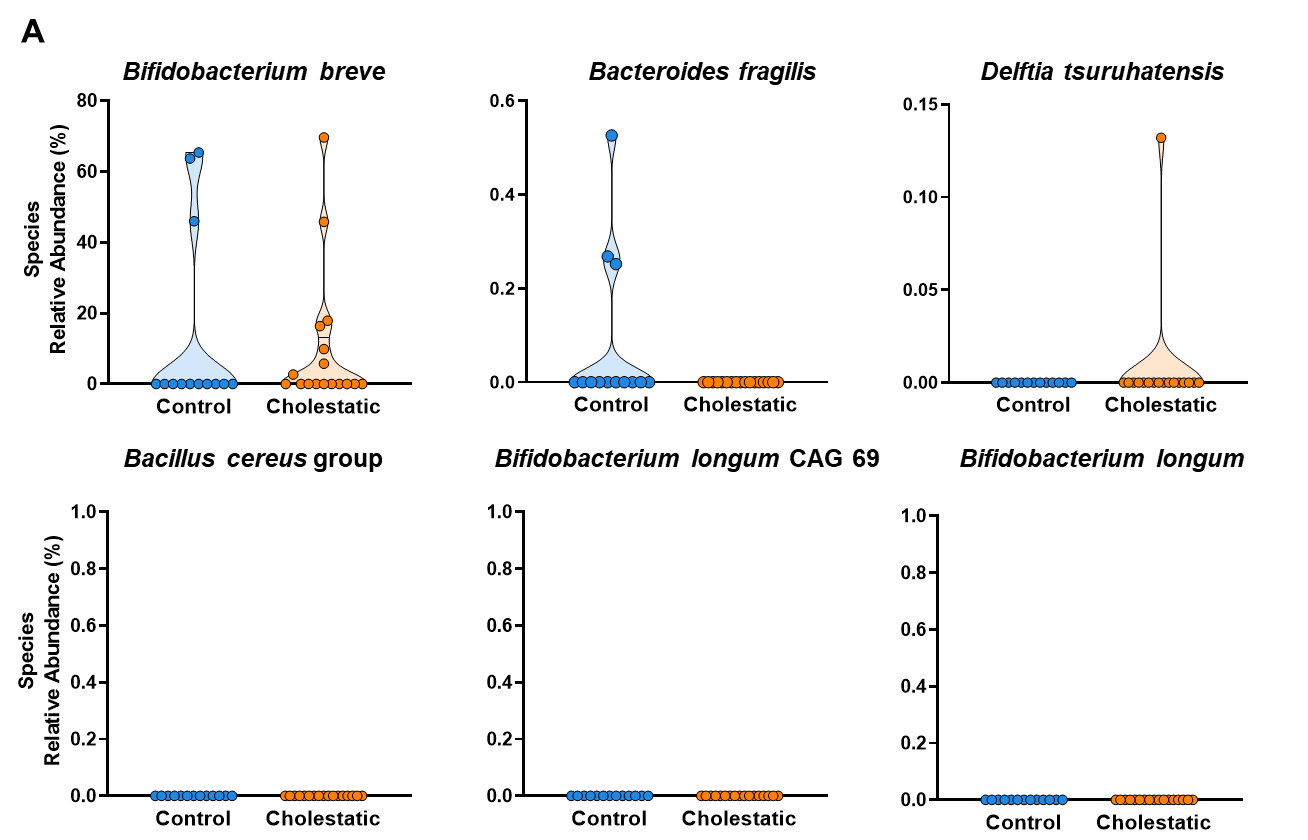


Figure S6. Relative abundance of BSH-carrying bacteria identified in dataset for preterm control (n = 13) and cholestatic (n = 17) infants at 32-40 weeks PMA. See also Figure 2e.


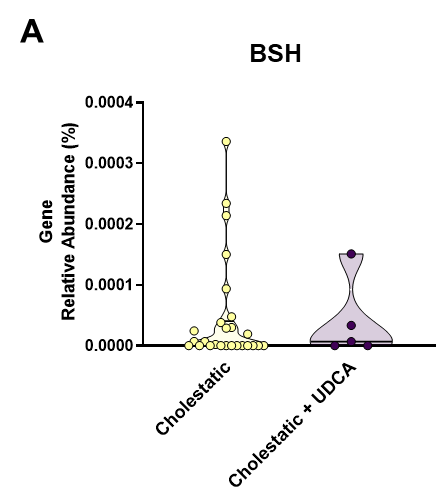


Figure S7. The relative abundance of the BSH gene is unchanged with UDCA treatment (n = 5-20). BSH, bile salt hydrolase; UDCA, ursodeoxycholic acid.

|  | Control  n = 12 | Cholestatic  n = 12 | p-value |
| --- | --- | --- | --- |
| Hospital length of stay (d), *median ± IQR* ^a^ | 109 ± 65.6 | 156 ± 62.7 | 0.099 |
| Weight at 36 wk PMA (g), *mean ± SD* ^b^ | 2100 ± 306 | 1835 ±380 | 0.082 |
| Length at 36 wk PMA (cm), *mean ± SD* ^a^ | 42.7 ± 2.9 | 41.7 ± 3.4 | 0.251 |
| Head circumference at 36 wk PMA (cm), *mean ± SD* ^b^ | 30.3 ± 1.4 | 28.6 ± 2.0 | 0.037* |
| Weight velocity (g/d), *mean ± SD* ^b^ | 19.0 ± 4.1 | 15.5 ± 5.4 | 0.097 |
| Length velocity (cm/wk), *mean ± SD* ^a^ | 0.94 ± 0.17 | 0.80 ± 0.26 | 0.149 |
| Head circumference velocity (cm/wk), *mean ± SD* ^b^ | 0.76 ± 0.140 | 0.63 ± 0.11 | 0.018* |
| Days to 140 mL/kg·d, *median ± IQR* ^b^ | 14.5 ± 4.8 | 46.7 ± 45.6 | 0.041* |
| Days of initial TPN during study, *median ± IQR* | 10.7 ± 3.7 | 34.6 ± 25.7 | 0.008** |
| Days *nil per os* > 12 h after feeds initiated, *median ± IQR* | 4.3 ± 8.4 | 23.2 ± 21.4 | 0.013* |
| BPD, *n (%)* ^b^ | 6 (50) | 8 (72.7) | 0.400 |
| NEC Stage ≥ IIA, *n (%)* | 0 (0) | 6 (50) | 0.014* |
| SIP, *n (%)* | 1 (8.3) | 4 (33.3) | 0.317 |
| Late-onset sepsis, *n (%)* | 2 (16.7) | 2 (16.7) | 1.000 |
| Death, *n (%)* | 0 (0.0) | 2 (16.7) | 0.478 |

Table S2: Clinical outcomes.

Values are mean ± SD (p-value from t-test), median ± IQR (p-value from Mann-Whitney test), or n (%) (p-value from Fisher’s exact test). BPD, bronchopulmonary dysplasia; NEC, necrotizing enterocolitis; PMA, post-menstrual age; SIP, spontaneous intestinal perforation; TPN, total parenteral nutrition. ** P < 0.01; * P < 0.05.

^a^ N = 10 in cholestatic cohort. Patients deceased before measurement (hospital length of stay). One patient removed from length measurements due to femur fracture.

^b^ N = 11 in cholestatic cohort. Patient deceased before measurement.

|  | Control  n = 12 | Cholestatic Untreated  n = 7 | Cholestatic  + UDCA  n = 5 | p-value |
| --- | --- | --- | --- | --- |
| Gestational age (wks), *mean ± SD* | 27.2 ± 1.8 | 28.0 ± 2.0 | 26.3 | 0.290 |
| Birth weight (g), *mean ± SD* | 968.2 ± 287.5 | 961.0 ± 264.7 | 872.0 ± 180.3 | 0.781 |
| Male, *n (%)* | 6 (50.0) | 4 (57.1) | 2 (40.0) | 1.000 |
| Delivery by C-section, *n (%)* | 11 (91.7) | 5 (71.4) | 4 (80.0) | 0.507 |
| SGA at birth, *n (%)* | 1 (8.3) | 3 (42.9) | 0 (0.0) | 0.097 |
| Mother’s milk (>90%), *n (%)* | 5 (41.7) | 5 (41.7) | 2 (40.0) | 1.000 |
| Received prophylactic indomethacin, *n (%)* | 5 (41.7) | 1 (14.3) | 2 (40.0) | 0.523 |
| Received surfactant, *n (%)* | 11 (91.7) | 5 (71.4) | 4 (80.0) | 0.507 |
| Apgar Score – 5 min, *median ± IQR* | 8.0 ± 1.3 | 8.0 ± 1.0 | 8.0 ± 2.0 | 0.931 |
| Received 2 doses of steroids, (*n %)* | 11 (91.7) | 5 (71.4) | 3 (60.0) | 0.317 |
| Days of antibiotics in first 14 d of life, *median ± IQR* | 3.0 ± 3.0 | 14.0 ± 6.5 | 5.0 ± 3.0 | 0.357 |
| Maternal antibiotic use, *n (%)* | 12 (100) | 7 (100) | 5 (100) | 1.000 |
| Preterm premature rupture of membranes, *n (%)* | 4 (33.3) | 1 (14.3) | 1 (20) | 0.828 |
| Maternal sepsis, n *(%)* | 1 (8.3) | 1 (14.3) | 1 (20) | 0.771 |

Table S3: Baseline demographics of patient population subset by treatment group.

Values are mean ± SD (p-value from one way ANOVA), median ± IQR (p-value from Kruskal-Wallis test), or n (%) (p-value from Fisher’s exact test). SGA, small for gestational age.

|  | 0-1 WOL | 2-3 WOL | 4+ WOL | p-value |
| --- | --- | --- | --- | --- |
| Observed OTUs | 10 ± 7 | 11 ± 9.5 | 14 ± 8 | 0.0559 |

Table S4. Alpha diversity by week of life in control preterm neonates (n = 18-25), Figure 1a.

Values are median ± IQR (p-value from Kruskal-Wallis test). OTUs, operational taxonomic units; WOL, week of life.

|  | 25-28 Weeks PMA | 29-30 Weeks PMA | 32-40 Weeks PMA | FDR global adjusted p |
| --- | --- | --- | --- | --- |
| *Clostridium perfringens* | 0.0 ± 0.2 | 0.0 ± 0.0 | 4.33 ± 7.75 | 0.010516 |
| *Staphylococcus epidermidis* | 19.89 ± 75.9 | 0.051 ± 8.03 | 0.0045 ± 0.50 | 0.02915 |
| *Gemella sanguinis* | 0.042 ± 0.005 | 0.0 ± 0.02 | 0.0 ± 0.0009 | 0.0396 |
| Secondary bile acid biosynthesis | 0.0 ± 0.0 | 0.0 ± 0.05 | 0.21 ± 1.46 | 0.00125121 |

Table S5. Abundances of significantly altered bacteria/pathways in Figures 1c and 1e (n = 13-31).

Values are median ± IQR (FDR global p-value from Kruskal-Wallis test). PMA, post-menstrual age.

| Pathway | LDA Score |
| --- | --- |
| Secondary bile acid biosynthesis | 3.64329532471 |
| Valine, leucine, and isoleucine degradation | -3.57077239929 |
| Tryptophan metabolism | -3.37743099134 |
| *Staphylococcus aureus* infection | -3.28703352046 |

Table S6. LEfSe results of significant metagenomic pathways in control preterm neonates 25-28 weeks PMA vs 32-40 weeks PMA (n = 20-31), Figure 1d.

LEfSe, linear discriminant analysis effect size; LDA, linear discriminant analysis; PMA, post-menstrual age.

| Sample | Coordinate | PMA |
| --- | --- | --- |
| FA-24-02-PP14 | 0.012327373 | 32.85714286 |
| FA-24-03-PP14 | -0.193290936 | 40 |
| FA-12-01-PP14 | -0.39432793 | 27.28571429 |
| FA-12-02-PP14 | -0.119155537 | 28 |
| FA-12-04-PP14 | -0.014028807 | 32 |
| 80-001-PP14 | -0.284774282 | 28.85714286 |
| 80-002-PP14 | -0.093151724 | 31 |
| 80-003-PP14 | 0.476337684 | 31.57142857 |
| 80-004-PP14 | 0.442074768 | 32.28571429 |
| 80-005-PP14 | 0.517358364 | 33.28571429 |
| FA-19-01-PP14 | -0.394121633 | 29.85714286 |
| FA-19-02-PP14 | -0.250618219 | 30.57142857 |
| FA-19-04-PP14 | 0.503404388 | 31.71428571 |
| FA-19-05-PP14 | 0.338795017 | 32.71428571 |
| FA-19-06-PP14 | 0.480166041 | 33.85714286 |
| FA-19-07-PP14 | 0.454259693 | 38.85714286 |
| 69-001-PP14 | -0.394846937 | 30.42857143 |
| 69-002-PP14 | -0.166758454 | 32.14285714 |
| 69-003-PP14 | 0.052217715 | 33.14285714 |
| 69-004-PP14 | 0.024453331 | 34.42857143 |
| 69-005-PP14 | 0.037141117 | 35.28571429 |
| 120-001-PP14 | -0.383059531 | 26.85714286 |
| 120-002-PP14 | -0.394535065 | 27 |
| 120-003-PP14 | -0.394579769 | 28.14285714 |
| 120-004-PP14 | -0.394839109 | 29 |
| 120-005-PP14 | -0.337646238 | 30.14285714 |
| 120-006-PP14 | -0.105986073 | 31 |
| 103-001-PP14 | 0.036985524 | 26 |
| 103-003-PP14 | 0.033707555 | 27.57142857 |
| 103-004-PP14 | 0.036964091 | 28.42857143 |
| 103-005-PP14 | -0.005032138 | 29.42857143 |
| 103-006-PP14 | 0.02627715 | 30.57142857 |
| 104-002-PP14 | -0.394375979 | 28.28571429 |
| 104-003-PP14 | -0.387141762 | 29.28571429 |
| 104-004-PP14 | -0.073777454 | 30.14285714 |
| 104-005-PP14 | -0.187488923 | 31.14285714 |
| FA-9-01-PP14 | 0.52501613 | 25.71428571 |
| FA-9-02-PP14 | 0.55600269 | 26.71428571 |
| FA-9-03-PP14 | 0.489122864 | 27.71428571 |
| FA-9-04-PP14 | 0.2774976 | 29 |
| FA-9-05-PP14 | 0.560892111 | 30.71428571 |
| FA-9-06-PP14 | 0.557229567 | 33.57142857 |
| 125-003-PP14 | 0.021024975 | 27.42857143 |
| 110-001-PP14 | -0.362912087 | 33.85714286 |
| 110-002-PP14 | -0.392769954 | 34.42857143 |
| 08-001-PP14 | 0.021190445 | 30.42857143 |
| 08-002-PP14 | 0.177353685 | 31.71428571 |
| 08-003-PP14 | 0.084071438 | 32.42857143 |
| 08-004-PP14 | 0.0162860666 | 34.42857143 |
| 102-001-PP14 | -0.035687734 | 29.28571429 |
| 102-002-PP14 | 0.010089776 | 29.57142857 |
| 102-003-PP14 | 0.00956108 | 30.42857143 |
| 102-004-PP14 | 0.01067785 | 30.85714286 |
| 102-005-PP14 | 0.001393573 | 31.85714286 |
| 102-006-PP14 | -0.022018443 | 32.85714286 |
| 102-007-PP14 | -0.002615292 | 33.85714286 |
| 123-001-PP14 | 0.021190445 | 28.85714286 |
| 123-002-PP14 | -0.394503634 | 29 |
| 31-002-PP14 | -0.17053022 | 28 |
| 31-003-PP14 | -0.034641445 | 28.28571429 |
| 31-004-PP14 | -0.029935838 | 29.28571429 |
| 31-005-PP14 | -0.032678635 | 30.71428571 |
| 31-006-PP14 | -0.034502772 | 31.14285714 |
| 31-007-PP14 | -0.039040678 | 32.14285714 |
| 33-001-PP14 | -0.254422574 | 27 |
| 33-002-PP14 | -0.069547857 | 28 |
| 33-004-PP14 | -0.090085637 | 30.42857143 |
| 33-005-PP14 | -0.11239512 | 31.28571429 |
| 46-001-PP14 | -0.060061644 | 25.57142857 |
| 46-002-PP14 | -0.252284448 | 25.85714286 |
| 46-003-PP14 | -0.397243938 | 26.71428571 |
| 46-004-PP14 | -0.239472376 | 27.71428571 |
| 46-005-PP14 | 0.521910476 | 28.85714286 |
| 46-006-PP14 | 0.53251771 | 29.85714286 |
| 59-001-PP14 | -0.21812695 | 26.42857143 |
| 59-002-PP14 | 0.046477995 | 28 |
| 59-003-PP14 | 0.085505011 | 28.71428571 |
| 59-004-PP14 | 0.153970666 | 29.42857143 |
| 59-005-PP14 | 0.163543393 | 30.42857143 |
| 61-001-PP14 | -0.411243415 | 27 |
| 61-002-PP14 | -0.12184218 | 27.85714286 |
| 61-003-PP14 | -0.259295995 | 30.28571429 |
| 61-004-PP14 | -0.178556043 | 30.85714286 |
| 64-001-PP14 | -0.360368517 | 28 |
| 64-002-PP14 | -0.127255135 | 28.14285714 |
| 64-003-PP14 | -0.385361161 | 28.28571429 |
| 64-004-PP14 | -0.395457367 | 28.42857143 |
| 64-005-PP14 | -0.194394856 | 30.14285714 |
| 64-007-PP14 | -0.086454439 | 31.42857143 |
| 70-001-PP14 | -0.298141204 | 30.71428571 |
| 70-002-PP14 | -0.039168576 | 31.71428571 |
| 70-003-PP14 | 0.026088985 | 33 |
| 70-004-PP14 | 0.040249637 | 34.28571429 |
| 70-005-PP14 | -0.341899162 | 35.14285714 |
| 81-002-PP14 | 0.537466722 | 30.14285714 |
| 81-003-PP14 | 0.538098878 | 30.28571429 |
| 81-004-PP14 | 0.528842652 | 30.42857143 |
| 81-005-PP14 | 0.521858845 | 31.42857143 |
| 81-006-PP14 | 0.502187988 | 32.42857143 |
| 81-007-PP14 | 0.519067439 | 33.57142857 |
| 81-008-PP14 | 0.530822218 | 34.57142857 |
| 94-002-PP14 | -0.299107779 | 29.57142857 |
| 94-003-PP14 | -0.244884278 | 29.71428571 |
| 94-004-PP14 | 0.091405006 | 30.14285714 |
| 94-005-PP14 | 0.468411959 | 30.85714286 |
| 94-006-PP14 | 0.519422312 | 31.85714286 |
| 94-007-PP14 | 0.395607355 | 32.85714286 |
| 94-008-PP14 | 0.40679398 | 33.85714286 |
| 99-001-PP14 | -0.393781805 | 26.14285714 |
| 99-002-PP14 | -0.394478848 | 26.28571429 |
| 99-003-PP14 | -0.153617967 | 27.28571429 |
| 99-005-PP14 | 0.000756353 | 29.28571429 |
| 99-006-PP14 | -0.008814341 | 30.57142857 |

Table S7. PC1 coordinates of metagenomic data vs PMA (n = 49-64), Figure 2a.

PMA, post-menstrual age.

| Pathway | LDA Score |
| --- | --- |
| Secondary bile acid biosynthesis | 3.37361491191 |
| Protein export | 3.10630215671 |
| Methane metabolism | 2.77425527126 |
| Neuroactive-ligand-receptor interaction | 2.44209126572 |
| Glycosaminoglycan degradation | 2.43370971426 |
| Nitrotoluene degradation | -2.01511438197 |
| Carotenoid biosynthesis | -2.53952480407 |
| Propanoate metabolism | -3.01425218698 |
| Tryptophan metabolism | -3.38760819521 |
| TCA cycle | -3.48197339215 |

Table S8. LEfSe results comparing significantly altered metagenomic pathways between control and cholestatic infants 32-40 weeks PMA (n = 13-17), Figure 2b.

PMA, post-menstrual age.

|  | Control | Cholestatic | p-value |
| --- | --- | --- | --- |
| Secondary bile acid biosynthesis, *median ± IQR* | 0.21 ± 1.5 | 0.10 ± 0.53 | 0.0385 |
| BSH gene, *median ± IQR* | 0.21 ± 1.5 | 0.10 ± 0.53 | 0.0385 |
| *Clostridium perfringens, median ± IQR* | 4.33 ± 7.75 | 0 ± 0.48 | 0.0008 |
| BSH activity – glycine, *mean ± SD* | 94.57 ± 19.59 | 23.51 ± 34.65 | 0.0037 |
| BSH activity – taurine, *mean ± SD* | 46.43 ± 18.67 | 20.20 ± 18.05 | 0.1155 |
| Unconjugated bile acid, *median ± IQR* | 26.99 ± 132.4 | 3.676 ± 32.4 | 0.0099 |
| Conjugated bile acid, *median ± IQR* | 2.83 ± 5.19 | 1.69 ± 6.74 | 0.4977 |

Table S9. Tabular results of Figure 2c-h (n = 5-18).

Values are mean ± SD (p-value from t-test) or median ± IQR (p-value from Mann-Whitney test). BSH, bile salt hydrolase.

| Sample | PC1 | PC2 |
| --- | --- | --- |
| 08-004-PP14 | 0.124104 | 0.000524 |
| 103-004-PP14 | -0.00725 | 0.121268 |
| 103-005-PP14 | -0.01034 | 0.014771 |
| 103-006-PP14 | -0.01447 | 0.123721 |
| 104-004-PP14 | 0.084518 | 0.002254 |
| 104-005-PP14 | -0.00631 | -0.00274 |
| 110-001-PP14 | 0.117007 | 0.020974 |
| 110-002-PP14 | 0.122937 | 0.007077 |
| 120-003-PP14 | 0.097738 | 0.018939 |
| 120-004-PP14 | -0.01052 | -0.00189 |
| 120-005-PP14 | -0.01308 | 0.016276 |
| 120-006-PP14 | 0.056389 | 0.001065 |
| 125-003-PP14 | 0.124647 | 0.001442 |
| 69-002-PP14 | 0.138306 | -0.00043 |
| 80-002-PP14 | 0.142946 | 0.001489 |
| 80-003-PP14 | -0.00537 | 0.166419 |
| 80-004-PP14 | -0.00503 | 0.140443 |
| 80-005-PP14 | 0.00016 | 0.146427 |
| FA-12-01-PP14 | 0.115223 | -0.02602 |
| FA-12-02-PP15 | 0.121694 | 0.002177 |
| FA-12-03-PP14 | 0.12409 | -0.00175 |
| FA-12-04-PP15 | 0.0209 | 0.133996 |
| FA-12-05-PP14 | -0.00515 | -0.00284 |
| FA-19-01-PP14 | 0.139288 | -0.0033 |
| FA-19-02-PP14 | 0.056214 | -0.00953 |
| FA-19-04-PP14 | 0.117951 | 0.006913 |
| FA-19-05-PP14 | 0.116503 | 0.007516 |
| FA-19-06-PP14 | -0.00549 | 0.166531 |
| FA-19-07-PP14 | -0.01196 | 0.005521 |
| FA-19-08-PP15 | -0.01205 | 0.010456 |
| FA-24-01-PP13 | 0.118311 | -0.00465 |
| FA-24-02-PP14 | -0.00555 | 0.166984 |
| FA-9-03-PP14 | -0.00577 | 0.161759 |
| FA-9-04-PP14 | 0.126715 | 0.019911 |
| FA-9-05-PP14 | 0.070195 | 0.117148 |
| 08-001-PP14 | 0.141385 | -0.01619 |
| 08-002-PP14 | 0.089573 | 0.003402 |
| 08-003-PP14 | -0.00814 | 0.14758 |
| 103-001-PP14 | 0.121952 | -0.0238 |
| 103-003-PP14 | 0.056022 | 0.033272 |
| 104-002-PP14 | 0.112014 | -0.02127 |
| 104-003-PP14 | 0.101044 | -0.00333 |
| 120-001-PP14 | 0.128685 | -0.02196 |
| 120-002-PP14 | 0.13827 | -0.00791 |
| 69-001-PP14 | 0.131783 | -0.01782 |
| 69-003-PP14 | 0.124452 | -0.00169 |
| 69-004-PP14 | -0.00318 | 0.162589 |
| 69-005-PP14 | -0.00483 | 0.15471 |
| 80-001-PP14 | 0.103663 | -0.02073 |
| FA-24-03-PP14 | 0.017603 | 0.117077 |
| FA-9-01-PP14 | 0.079853 | 0.132061 |
| FA-9-02-PP14 | 0.015646 | 0.146121 |
| FA-9-06-PP14 | 0.12309 | 0.051967 |
| 102-001-PP14 | 0.13242 | -0.01807 |
| 102-002-PP14 | 0.113858 | -0.0245 |
| 102-003-PP14 | 0.097513 | -0.01674 |
| 102-004-PP14 | 0.123436 | 0.022053 |
| 102-005-PP14 | -0.00779 | 0.16337 |
| 102-006-PP14 | -0.00636 | 0.165226 |
| 102-007-PP14 | -0.00556 | 0.161513 |
| 123-001-PP14 | 0.120016 | -0.02407 |
| 123-002-PP14 | 0.120398 | -0.02432 |
| 123-003-PP15 | 0.132579 | 0.012856 |
| 123-004-PP16 | 0.135069 | 0.008204 |
| 123-005-PP17 | 0.056775 | 0.073236 |
| 123-006-PP18 | 0.129278 | 0.035036 |
| 123-007-PP19 | 0.118024 | 0.063776 |
| 123-008-PP20 | 0.018548 | 0.160462 |
| 31-001-PP13 | 0.114958 | -0.02347 |
| 31-002-PP14 | 0.127465 | 0.000768 |
| 31-003-PP14 | 0.120318 | -0.01841 |
| 31-004-PP14 | 0.107365 | -0.00255 |
| 31-005-PP14 | 0.120113 | 0.002865 |
| 31-006-PP14 | 0.074799 | 0.109316 |
| 31-007-PP14 | 0.069346 | 0.121635 |
| 33-001-PP14 | 0.118305 | -0.00981 |
| 33-002-PP14 | 0.127884 | 0.001063 |
| 33-004-PP14 | 0.104237 | -0.00444 |
| 33-005-PP14 | 0.090137 | 0.00985 |
| 46-001-PP14 | 0.125609 | -0.01032 |
| 46-002-PP14 | 0.137628 | -0.00727 |
| 46-003-PP14 | 0.094943 | -0.01495 |
| 46-004-PP14 | 0.112147 | 0.069275 |
| 46-005-PP14 | -0.00339 | 0.166882 |
| 46-006-PP14 | -0.00526 | 0.153985 |
| 59-001-PP14 | 0.100447 | -0.01187 |
| 59-002-PP14 | -0.00841 | 0.166658 |
| 59-003-PP14 | -0.00827 | 0.165657 |
| 59-004-PP14 | -0.00813 | 0.166473 |
| 59-005-PP14 | -0.00776 | 0.164788 |
| 61-001-PP14 | 0.116438 | -0.02211 |
| 61-002-PP14 | 0.112138 | 0.005279 |
| 61-003-PP14 | 0.139783 | -0.00068 |
| 61-004-PP14 | 0.119535 | 0.006103 |
| 64-001-PP14 | 0.109606 | -0.02279 |
| 64-002-PP14 | 0.103292 | -0.02087 |
| 64-003-PP14 | 0.08048 | -0.02087 |
| 64-004-PP14 | 0.117867 | -0.01619 |
| 64-005-PP14 | 0.025028 | 0.105487 |
| 64-007-PP14 | -0.0045 | 0.146057 |
| 70-001-PP14 | 0.141294 | -0.00854 |
| 70-002-PP14 | 0.128455 | -0.0006 |
| 70-003-PP14 | 0.004066 | 0.164357 |
| 70-004-PP14 | -0.00685 | 0.167752 |
| 70-005-PP14 | -0.00619 | 0.117457 |
| 81-002-PP14 | 0.131717 | -0.01725 |
| 81-003-PP14 | 0.118919 | -0.01915 |
| 81-004-PP14 | 0.099584 | -0.0203 |
| 81-005-PP14 | 0.110382 | -0.00315 |
| 81-006-PP14 | -0.00588 | 0.115214 |
| 81-007-PP14 | -0.00616 | 0.122584 |
| 81-008-PP14 | -0.00583 | 0.137501 |
| 94-002-PP14 | 0.104707 | 0.022835 |
| 94-003-PP14 | 0.006453 | 0.047252 |
| 94-004-PP14 | -0.00664 | 0.150682 |
| 94-005-PP14 | -0.00651 | 0.161633 |
| 94-006-PP14 | -0.00777 | 0.14479 |
| 94-007-PP14 | -0.00989 | 0.158885 |
| 94-008-PP14 | -0.00665 | 0.159704 |
| 99-001-PP14 | 0.10896 | -0.0213 |
| 99-002-PP14 | 0.112314 | -0.02024 |
| 99-003-PP14 | -0.00537 | 0.133198 |
| 99-005-PP14 | -0.00688 | 0.167334 |
| 99-006-PP14 | -0.00665 | 0.132279 |

Table S10. Principal coordinates of bile acid profiles for all neonates (n = 124), Figure 4a.

PC, principal coordinate.

|  | Control | Cholestatic | Cholestatic + UDCA | p-value |
| --- | --- | --- | --- | --- |
| Total Fecal BA | 27.4 ± 117.1 | 4.1 ± 8.4 | 35 ± 84.1 | 0.005 |

Table S11. Tabular data of Figure 4b comparing total fecal BAs in control, UDCA treated cholestatic, and untreated cholestatic infants (n = 7-20).

Values are median ± IQR (p-value from Kruskal-Wallis test). BA, bile acid; UDCA, ursodeoxycholic acid.

|  | Cholestatic | Cholestatic + UDCA | p-value |
| --- | --- | --- | --- |
| Total Fecal BA | 4.1 ± 8.4 | 35 ± 84.1 | 0.005 |
| UDCA | 0.07 ± 0.12 | 34.0 ± 79.57 | <0.0001 |
| Actinobacteria | 3.2 ± 10.1 | 0.000 ± 0.002 | 0.051532 |
| Firmicutes | 16.8 ± 69.4 | 100 ± 61.6 | 0.024102 |
| Proteobacteria | 73.5 ± 87.3 | 0.0 ± 59.4 | 0.045410 |
| *Clostridium perfringens* | 0.0 ± 0.0 | 0.63 ± 7.34 | 0.036 |
| *Bifidobacterium breve* | 0.0 ± 6.0 | 0.0 ± 0.0 | 0.1337 |

Table S12. Tabular data of Figure 4 comparing bile acid and microbiome communities in UDCA treated vs untreated cholestatic neonates (n = 5-20).

Values are median ± IQR (p-value from Mann-Whitney test or Kruskal-Wallis test). BA, bile acid; UDCA, ursodeoxycholic acid.
